# A Novel Glycoproteomics Workflow Reveals Dynamic O-GlcNAcylation of COPγ1 as a Candidate Regulator of Protein Trafficking

**DOI:** 10.3389/fendo.2018.00606

**Published:** 2018-10-15

**Authors:** Nathan J. Cox, Peter M. Luo, Timothy J. Smith, Brittany J. Bisnett, Erik J. Soderblom, Michael Boyce

**Affiliations:** ^1^Department of Biochemistry, Duke University School of Medicine, Durham, NC, United States; ^2^Proteomics and Metabolomics Core Facility, Center for Genomic and Computational Biology, Duke University, Durham, NC, United States

**Keywords:** O-GlcNAc, glycoproteomics, SILAC, click chemistry, COPI vesicle trafficking, protein secretion

## Abstract

O-linked β-N-acetylglucosamine (O-GlcNAc) is an abundant and essential intracellular form of protein glycosylation in animals and plants. In humans, dysregulation of O-GlcNAcylation occurs in a wide range of diseases, including cancer, diabetes, and neurodegeneration. Since its discovery more than 30 years ago, great strides have been made in understanding central aspects of O-GlcNAc signaling, including identifying thousands of its substrates and characterizing the enzymes that govern it. However, while many O-GlcNAcylated proteins have been reported, only a small subset of these change their glycosylation status in response to a typical stimulus or stress. Identifying the functionally important O-GlcNAcylation changes in any given signaling context remains a significant challenge in the field. To address this need, we leveraged chemical biology and quantitative mass spectrometry methods to create a new glycoproteomics workflow for profiling stimulus-dependent changes in O-GlcNAcylated proteins. In proof-of-principle experiments, we used this new workflow to interrogate changes in O-GlcNAc substrates in mammalian protein trafficking pathways. Interestingly, our results revealed dynamic O-GlcNAcylation of COPγ1, an essential component of the coat protein I (COPI) complex that mediates Golgi protein trafficking. Moreover, we detected 11 O-GlcNAc moieties on COPγ1 and found that this modification is reduced by a model secretory stress that halts COPI trafficking. Our results suggest that O-GlcNAcylation may regulate the mammalian COPI system, analogous to its previously reported roles in other protein trafficking pathways. More broadly, our glycoproteomics workflow is applicable to myriad systems and stimuli, empowering future studies of O-GlcNAc in a host of biological contexts.

## Introduction

O-linked β-N-acetylglucosamine (O-GlcNAc) is a major, dynamic post-translational modification (PTM), added by O-GlcNAc transferase (OGT) and removed by O-GlcNAcase (OGA) from serine and threonine residues of intracellular proteins (1–7). O-GlcNAc is broadly conserved among animals, plants and other organisms, and O-GlcNAcylation controls a wide range of cellular functions, such as nutrient sensing, metabolism and gene expression (1–8). Importantly, aberrant O-GlcNAc cycling is also implicated in numerous human diseases, including cancer (2, 9–12), diabetes (13–16), cardiac dysfunction (17–20), and neurodegeneration (21–24).

Despite this broad pathophysiological significance, major questions about O-GlcNAc signaling remain. For example, O-GlcNAcylation regulates diverse cellular processes and modifies thousands of intracellular proteins, but only a small fraction of substrates change their glycosylation status in response to any given signal or condition (1–4, 25, 26). A central challenge in the field is to identify the most functionally relevant O-GlcNAc changes in response to a stimulus of interest. However, because O-GlcNAc is a transient and sub-stoichiometric PTM, it can be difficult to study with traditional molecular biology or genetics alone.

To address this challenge, we previously reported a two-step chemical biology method to tag and purify O-GlcNAc substrates from live mammalian cells (27–31). Briefly, cells are first metabolically labeled with a peracetylated N-azidoacetylgalactosamine (GalNAz), a synthetic, azide-bearing monosaccharide that is non-toxic and cell-permeable (28, 32). GalNAz is accepted by sugar salvage and epimerase enzymes, resulting in the biosynthesis of a nucleotide-azidosugar, “UDP-GlcNAz,” which is used by OGT to install an “O-GlcNAz” moiety onto its native substrates (28). O-GlcNAz can then be tagged via chemical ligation to an alkyne-functionalized probe. Azides and alkynes engage in a copper-catalyzed [3+2] cycloaddition, often called “click chemistry,” that proceeds rapidly under biocompatible conditions (33–36). The click reaction between O-GlcNAz moieties and the alkyne probe provides exquisitely specific labeling of OGT substrates with useful handles (e.g., biotin, fluorophores) for downstream analysis (27–31). Because GalNAz treatment labels endogenous OGT substrates, it affords time-resolved tagging of O-GlcNAcylated proteins, without the need for a priori knowledge of their identities. We have previously used this strategy to dissect the functional role of O-GlcNAc in a variety of cell biological contexts (28–31).

We envisioned combining GalNAz labeling with quantitative proteomics to discover changes in O-GlcNAcylated proteins in response to physiological stimuli, stresses, or other cues. As a model cellular process for these proof-of-principle experiments, we selected protein trafficking. More than a third of mammalian proteins transit the secretory system to localize to, and recycle from, specific subcellular locations, including the endoplasmic reticulum (ER), Golgi, plasma membrane, endosomes, lysosomes, and the extracellular space (37–39). In most instances, dedicated protein machinery effects the formation and trafficking of vesicles between discrete locations, as in clathrin-mediated endocytosis from the plasma membrane to endosomes (40), coat protein complex II (COPII)-facilitated transport from the ER to Golgi (41–46), and COPI-mediated trafficking among the Golgi cisternae and from the Golgi to the ER (47–49). Properly regulated protein trafficking is critical for cell and tissue physiology, particularly in professional secretory cell types and organs, including the endocrine system. Indeed, protein trafficking is essential in all eukaryotes and is dysregulated in a wide range of human diseases (44–46).

While the fundamental biochemical steps of vesicle assembly are relatively well understood for some systems (e.g., clathrin, COPII, and COPI), much less is known about how vertebrate cells dynamically adjust trafficking activity in response to developmental cues, fluctuating signals, metabolic demands, or stress (44–46). Interestingly, however, several studies have implicated O-GlcNAc in regulating multiple protein trafficking pathways. For example, key COPII proteins are O-GlcNAcylated (28, 50–53), and we recently demonstrated that specific glycosylation sites on Sec23A, a core COPII protein, are required for its ability to mediate collagen trafficking in both cultured human cells and developing vertebrate embryos (54). Other studies have indicated a role for O-GlcNAc in regulating synaptic vesicle trafficking and clathrin-mediated endocytosis as well (55–63). Taken together, these reports suggest that O-GlcNAc may be a broad regulator of protein trafficking. However, the extent and functional effects of O-GlcNAcylation in mammalian trafficking pathways remain largely uncharacterized.

Here, we leverage GalNAz metabolic labeling and quantitative proteomics to create a novel workflow for identifying stimulus-induced changes in O-GlcNAcylated proteins. In a pilot experiment, we used this glycoproteomics workflow to investigate the role of O-GlcNAc in mammalian protein trafficking. Our results indicate that COPγ1, an essential component of the COPI complex, is dynamically O-GlcNAcylated on up to 11 distinct sites under control conditions but deglycosylated upon perturbation of protein secretion. Our study is the first report of COPI protein O-GlcNAcylation and suggests that O-GlcNAc may regulate mammalian intra-Golgi and/or retrograde Golgi-to-ER protein trafficking. More broadly, we expect that our glycoproteomics strategy will be readily extensible to a wide spectrum of experimental stimuli, conditions and systems beyond protein trafficking, permitting the study of O-GlcNAc function in diverse biological contexts.

## Materials and methods

### Chemical synthesis

Thiamet-G and Ac_4_GalNAz were synthesized as described (28, 64) by the Duke Small Molecule Synthesis Facility. All other chemicals were purchased from Sigma-Aldrich unless otherwise indicated.

### Cell culture

Ramos cells were cultured in Roswell Park Memorial Institute medium (RPMI) containing 10% fetal bovine serum (FBS), 100 units/ml penicillin, and 100 μg/ml streptomycin in 5% CO_2_ at 37°C. FL5.12 (parental N6 and XL4.1 lines) were cultured in RPMI containing 10% FBS, 100 units/ml penicillin, 100 μg/ml streptomycin, 55 μM β-mercaptoethanol, 2 mM L-glutamine, 10 mM HEPES and 500 pg/ml recombinant mouse IL-3 (eBioscience) in 5% CO_2_ at 37°C.

### Cell viability assays

Ten thousand Parental FL5.12 (N6) cells in 100 μl RPMI were seeded into clear-bottom 96-well plates and treated with a dose range of brefeldin A (BFA) for 4 or 24 h. Both MTS (Promega, CellTiter 96 AQueous Proliferation Assay) and ATP (Promega, CellTiter-Glo Luminescent Cell Viability Assay) assays were performed according to the manufacturer's instructions. Independent replicates were evaluated by a 2 × 2 analysis of variance (ANOVA), with BFA dose and treatment time as the independent factors. *Post-hoc* tests for differences between BFA doses and treatment times were conducted with Tukey's honestly significant difference (HSD) test using SAS/JMP software, Version 13.0.0 (SAS Institute Inc.). Significance was defined as *p* < 0.05 (two-tailed).

### Alkyne-biotin click reactions and affinity purification

Cells were treated with 100 μM GalNAz up to 24 h prior to harvesting. After harvesting, cells were lysed in click buffer (1% Triton X-100, 1% SDS, 150 mM NaCl, 20 mM Tris pH 7.4) supplemented with protease inhibitors, 5 μM PUGNAc and 50 μM UDP to inhibit hexosaminidases and OGT, respectively. Click reactions were assembled on an ice bucket. The following reaction components were added, in order, to the listed final concentration: protein sample, 5 mM sodium ascorbate, 25 μM alkyne-biotin, 100 μM Tris[(1-benzyl-1H-1,2,3-triazol-4-yl)methyl]amine (TBTA), 1 mM CuSO_4_. Reactions were mixed, rotated gently at room temperature for 1 h and then quenched by addition of 10 mM EDTA (final). For immediate analysis, SDS-PAGE sample buffer was added directly to reactions. For further processing and affinity purification, unreacted alkyne-biotin was removed by methanol-precipitation as follows. Reactions were mixed with ice-cold methanol (10:1 methanol:sample by volume). After mixing, samples were placed on dry ice or incubated at −80°C for 10 min to increase protein precipitation and then centrifuged at 17,000 g to pellet. Supernatants were removed and pellets were resuspended in methanol and placed on ice. This process was repeated a total of four times. After the final precipitation, the protein pellet was dissolved in 4 M guanidine in phosphate-buffered saline (PBS). Biotinylated proteins were captured from the samples by incubating overnight at 4°C with gentle rotation with NeutrAvidin beads (ThermoFisher, Pierce High Capacity NeutrAvidin Agarose). The following day, beads were washed three times with the following buffers, in order: 4 M guanidine in PBS, 5 M NaCl in H_2_O, 6 M urea in PBS, and 1% SDS in PBS. Captured proteins were eluted by boiling in 2X SDS-PAGE sample buffer. Reserved input samples in 4 M guanidine were buffer-exchanged into SDS-PAGE buffer via spin column (BioRad, Bio-Spin 6).

### Immunoblotting (IB)

IBs were performed via standard methods as previously described (54). The following primary antibodies were used: mouse monoclonal anti-tubulin (T6074, Sigma-Aldrich; 1:100,000), mouse monoclonal anti-biotin (B7653, Sigma-Aldrich, 1:2,000), mouse monoclonal anti-nucleoporin p62 (610498, BD Biosciences, 1:2,000), mouse monoclonal anti-COPγ1 (sc-393977, Santa Cruz Biotechnology, 1:1,000), mouse monoclonal anti-O-GlcNAc antibody 18B10 (MA1-038, ThermoFisher; 1:1,000), mouse monoclonal anti-O-GlcNAc antibody RL2 (SC-59624, Santa Cruz Biotech; 1:500). The following secondary antibody was used: goat anti-mouse IgG (1030-05, horseradish peroxidase (HRP)-conjugated, SouthernBiotech; 1:10,000).

### SILAC labeling

RPMI 1640 medium lacking L-lysine and L-arginine (ThermoFisher) was supplemented with 10% dialyzed and heat-inactivated FBS (Corning), 1% penicillin/streptomycin and amino acids. “Heavy” medium was supplemented with 12.5 mg ^13^${\text{C}}_{6}^{15}$N_4_-arginine, 12.5 mg ^13^${\text{C}}_{6}^{15}$N_2_-lysine, and 5 mg proline per 500 ml. “Light” medium was supplemented with 12.5 mg arginine, 12.5 mg lysine, and 5 mg proline per 500 ml. Proline supplementation prevents conversion of arginine to proline (65). XL4.1 cells were passaged for at least 7 doublings in either heavy or light SILAC medium to achieve >99% isotope incorporation. Isotope incorporation was verified via MS at the Duke Proteomics Facility. Full proteomics data are available as Excel files in the Supplemental Material.

### Subcellular fractionation

Cells were washed once with cold PBS and resuspended in 5 ml of ice-cold Buffer A (1.5 mM MgCl_2_, 10 mM KCl, 10 mM HEPES, pH 7.9) supplemented with protease inhibitors, 5 μM PUGNAc and 50 μM UDP. Cells were lysed using a pre-chilled Dounce homogenizer and ~30 strokes with a tight pestle. Cell integrity was monitored using a hemocytometer. After Douncing, samples were centrifuged at 228 g for 5 min at 4°C, yielding a crude cytoplasmic fraction (supernatant) and a crude nuclear fraction (pellet). Crude nuclear fractions were resuspended in 3 ml of Buffer S1 (0.35 M sucrose, 0.5 mM MgCl_2_) supplemented with protease inhibitors, PUGNAc and UDP, layered over a cushion of Buffer S3 (0.88 M sucrose, 0.5 mM MgCl_2_) and centrifuged at 2,800 g for 10 min at 4°C to obtain a pure nuclear pellet. Crude cytoplasmic fractions were centrifuged at >400,000 g for 1 h at 4°C to obtain a pure cytoplasmic fraction (supernatant). Pure nuclear pellets were lysed in click buffer and pure cytoplasmic fractions were supplemented with appropriate concentrations of click buffer ingredients.

### Proteomic analysis of BFA-induced changes in O-GlcNacylated proteins

XL4.1 cells were seeded at 500,000 cells/ml the day before treatment. Cells were treated with 100 μM GalNAz alone for 2 h, then 500 ng/ml BFA or DMSO (vehicle) was added, and cells were harvested 4 h later. Heavy- and light-labeled cells were pooled 1:1, washed twice with cold PBS and fractionated as above. Protein amounts were quantified by BCA Assay (ThermoFisher) and 2 mg of nuclear or cytoplasmic protein was processed further. Alkyne-agarose beads (Click Chemistry Tools) were washed three times in click buffer. Protein samples were precleared with 150 μl bead volume of washed alkyne-agarose beads with gentle rotation for 2 h at room temperature. After preclearing, supernatants were removed and combined with 50 μl of equilibrated alkyne-agarose beads, 5 mM sodium ascorbate, 100 μM TBTA and 1 mM CuSO_4_. Reactions were rotated at room temperature for 2 h and then quenched by addition of 10 mM EDTA. Beads were washed sequentially with three 1 ml washes of each of the following: 1% SDS, 20 mM Tris pH 7.4; 1% SDS, 10 mM dithiothreitol (DTT), 20 mM Tris pH 7.4; 1X PBS; 8 M urea; 1X PBS; 6 M guanidine hydrochloride; 1X PBS; 5 M NaCl; 1X PBS; 10X PBS; 1X PBS; 20% isopropanol; 20% acetonitrile; 50 mM ammonium bicarbonate. Washed beads were stored at 4°C in 100 μl 50 mM ammonium bicarbonate until they were submitted for on-bead trypsin digestion, LC-MS/MS analysis and quantification at the Duke Proteomics Facility.

### Sample preparation and nano-flow liquid chromatography electrospray ionization tandem mass spectrometry (LC-MS/MS) analysis of SILAC samples

Samples immobilized on alkyne-agarose beads were washed three times with 50 mM ammonium bicarbonate, pH 8.0 and suspended in 30 μl 50 mM ammonium bicarbonate, pH 8.0 supplemented with 0.1% Rapigest SF surfactant (Waters). Samples were reduced with 5 mM DTT for 30 min at 70°C and free sulfhydryls were alkylated with 10 mM iodoacetamide for 45 min at room temperature. Proteolytic digestion was accomplished by the addition of 500 ng sequencing grade trypsin (Promega) directly to the beads with incubation at 37°C for 18 h. Supernatants were collected following a 2-min centrifugation at 1,000 rpm, acidified to pH 2.5 with trifluoroacetic acid and incubated at 60°C for 1 h to hydrolyze the remaining Rapigest. Insoluble hydrolyzed surfactant was cleared by centrifugation at 15,000 rpm for 5 min. Samples were dried using vacuum centrifugation and resuspended in 20 μl of 2% acetonitrile/0.1% formic acid. Two microliters of each sample was subjected to chromatographic separation on a Waters NanoAquity UPLC equipped with a 1.7 μm BEH130 C_18_ 75 μm I.D. X 250 mm reversed-phase column. The mobile phase consisted of (A) 0.1% formic acid in water and (B) 0.1% formic acid in acetonitrile. Following a 5 μl injection, peptides were trapped for 5 min on a 5 μm Symmetry C_18_ 180 μm I.D. X 20 mm column at 20 μl/minute in 99.9% A. The analytical column was held at 5% B for 5 min, then switched in-line and a linear elution gradient of 5% B to 40% B was performed over 90 min at 300 nl/minute. The analytical column was connected to a fused silica PicoTip emitter (New Objective) with a 10 μm tip orifice and coupled to a QExactive Plus mass spectrometer through an electrospray interface. The instrument was set to acquire a precursor MS scan from *m/z* 375–1,600 with *r* = 70,000 at *m/z* 400 and a target AGC setting of 1e6 ions. In a data-dependent mode of acquisition, MS/MS spectra of the 10 most abundant precursor ions were acquired at *r* = 17,500 at m/z with a target AGC setting of 5e4 ions. Max fill times were set to 60 ms for full MS scans and 60 ms for MS/MS scans with minimum MS/MS triggering thresholds of 5,000 counts. For all experiments, fragmentation occurred with a higher-energy collisional dissociation setting of 27% and a dynamic exclusion of 60 s were employed for previously fragmented precursor ions.

Raw LC-MS/MS data files were processed in Mascot distiller (Matrix Science) and then submitted to independent Mascot database searches (Matrix Science) against SwissProt (*Mus musculus* taxonomy) containing both forward and reverse entries of each protein. Search tolerances were 5 ppm for precursor ions and 0.02 Da for product ions using trypsin specificity with up to two missed cleavages. Carbamidomethylation (+57.0214 Da on Cys) was set as a fixed modification, whereas oxidation (+15.9949 Da on Met) and O-GlcNAcylation (+203 Da on Ser/Thr) were considered variable modifications. All searched spectra were imported into Scaffold (Proteome Software) and protein confidence thresholds were set using a Bayesian statistical algorithm based on the PeptideProphet and ProteinProphet algorithms, which yielded a peptide and protein false discovery rate (FDR) of 1%.

SILAC data were processed using Rosetta Elucidator as previously described (66–68) with the following modifications. Database searching in Mascot used a SwissProt mouse database (downloaded on 4/21/11) with an equal number of reverse entries, 5 ppm precursor and 0.02 Da product ion tolerances and variable modifications on Met (oxidation), Arg (+10), and Lys (+8). Data were annotated at a 1% peptide FDR using the PeptideTeller algorithm. Quantification of labeled pairs required that both members were identified.

### Immunoprecipitation (IP)

Cells were washed twice with cold PBS and lysed in IP lysis buffer (1% Triton X-100, 150 mM NaCl, 1 mM EDTA, 20 mM Tris-HCl pH 7.4) supplemented with protease inhibitors, 5 μM PUGNAc and 50 μM UDP. Lysates were probe-sonicated, cleared by centrifugation and quantified by BCA protein assay. IPs were performed on 1–5 mg total protein. Cleared lysates were adjusted to a final total protein concentration of ~1 mg/ml using IP lysis buffer. For every 1 mg of protein lysate used, 3 μg of mouse monoclonal anti-COPγ1 (sc-393977, Santa Cruz Biotechnology) antibody was added and rotated overnight at 4°C. The following day, 50 μl equilibrated protein A/G UltraLink Resin (ThermoFisher) was added to the lysate and rotated at room temperature for 1 h. Beads were washed three times with 1 ml of IP lysis buffer and then eluted in 2X SDS-PAGE sample buffer with boiling. Eluents were analyzed via IB.

### Cloning

The COPγ1-myc-6xHis construct was generated by amplifying the open reading frame of the human COPγ1 cDNA (Harvard PlasmID Repository) by PCR and ligating it into the *Hind*III and *Not*I sites of pcDNA4/myc-6xHis (Invitrogen) using standard methods.

### Transfections

293T cells plated at ~50% confluence were transfected the following day as previously described (54). In brief, 750 μl of prewarmed OPTI-MEM was placed into 1.5 ml tubes with 45 μl of TransIT-293 transfection reagent (Mirus), vortexed briefly, and incubated for 15 min at room temperature. Next, 15 μg of human COPγ1-myc-6xHis DNA was added to the tube, vortexed briefly, and incubated for 15 min at room temperature. After the final incubation, the mixture was added dropwise to the cells. Cells were harvested 48 h after transfection.

### Tandem purification of COPγ1-myc-6xHis

293T cells transfected with COPγ1-myc-6xHis were treated 8 h prior to harvest with 50 μM Thiamet-G and 4 mM glucosamine to enhance O-GlcNAcylation. Cells were harvested in cold PBS and lysed in IP lysis buffer supplemented with 0.1% SDS, protease inhibitors, 5 μM PUGNAc and 50 μM UDP. Lysates were probe sonicated, cleared by centrifugation, and quantified by BCA protein assay according to the manufacturer's instructions. Myc IPs were performed on ~100 mg of total protein for MS analysis. Cleared lysates were adjusted to a final protein concentration of 2 mg/ml using IP lysis buffer supplemented with 0.1% SDS, protease inhibitors, 5 μM PUGNAc, and 50 μM UDP. Three micrograms of mouse monoclonal anti-c-myc (9E10, BioLegend) per mg of total protein was added and rotated overnight at 4°C. The following day, 50 μl of washed protein A/G UltraLink Resin (53133, ThermoFisher) was added and the mixture was rotated at room temperature for 1 h. Beads were washed three times with 1 ml of IP lysis buffer with 0.1% SDS and eluted twice in 500 μl using Ni-NTA wash buffer (8 M urea, 300 mM NaCl, 1% Triton X-100, and 5 mM imidazole) with rotation at room temperature. The two 500 μl elutions were pooled, 50 μl of washed 6xHisPur Ni-NTA resin (88223, ThermoFisher) was added to the eluate and the mixture rotated for 2 h at room temperature. The Ni-NTA resin was washed three times with 1 ml of Ni-NTA wash buffer and eluted in 8 M urea plus 250 mM imidazole.

### LC-MS/MS analysis of COPγ1 O-GlcNacylation

Purified COPγ1-myc-6xHis was separated by SDS-PAGE and Coomassie-stained. Stained bands of the correct molecular weight were subjected to standard in-gel trypsin digestion (https://genome.duke.edu/sites/genome.duke.edu/files/In-gelDigestionProtocolrevised_0.pdf). Extracted peptides were lyophilized to dryness and resuspended in 12 μl of 0.2% formic acid/2% acetonitrile. Each sample was subjected to chromatographic separation on a Waters NanoAquity UPLC equipped with a 1.7 μm BEH130 C_18_ 75 μm I.D. X 250 mm reversed-phase column. The mobile phase consisted of (A) 0.1% formic acid in water and (B) 0.1% formic acid in acetonitrile. Following a 4 μl injection, peptides were trapped for 3 min on a 5 μm Symmetry C_18_ 180 μm I.D. X 20 mm column at 5 μl/minute in 99.9% A. The analytical column was then switched in-line and a linear elution gradient of 5% B to 40% B was performed over 60 min at 400 nl/minute. The analytical column was connected to a fused silica PicoTip emitter (New Objective, Cambridge, MA) with a 10 μm tip orifice and coupled to a QExactive Plus mass spectrometer (Thermo) through an electrospray interface operating in data-dependent acquisition mode. The instrument was set to acquire a precursor MS scan from m/z 350 to 1,800 every 3 s. In data-dependent mode, MS/MS scans of the most abundant precursors were collected following higher-energy collisional dissociation (HCD) fragmentation at an HCD collision energy of 27%. Within the MS/MS spectra, if any diagnostic O-GlcNAc fragment ions (m/z 204.0867, 138.0545, or 366.1396) were observed, a second MS/MS spectrum of the precursor was acquired with electron transfer dissociation (ETD)/HCD fragmentation using charge-dependent ETD reaction times and either 30 or 15% supplemental collision energy for ≥2+ precursor charge states. For all experiments, a 60-s dynamic exclusion was employed for previously fragmented precursor ions.

Raw LC-MS/MS data files were processed in Proteome Discoverer (Thermo Scientific) and then submitted to independent Mascot searches (Matrix Science) against a SwissProt database (human taxonomy) containing both forward and reverse entries of each protein (20,322 forward entries). Search tolerances were 5 ppm for precursor ions and 0.02 Da for product ions using semi-trypsin specificity with up to two missed cleavages. Both y/b-type HCD and c/z-type ETD fragment ions were allowed for interpreting all spectra. Carbamidomethylation (+57.0214 Da on C) was set as a fixed modification, whereas oxidation (+15.9949 Da on M) and O-GlcNAc (+203.0794 Da on S/T) were considered dynamic mass modifications. All searched spectra were imported into Scaffold (v4.3, Proteome Software) and scoring thresholds were set to achieve a peptide FDR of 1% using the PeptideProphet algorithm. When satisfactory ETD fragmentation was not obtained, HCD fragmentation was used to determine O-GlcNAc residue modification, using the number of HexNAcs identified in combination with the number of serines and threonines in the peptide.

## Results

**Figure 1:**
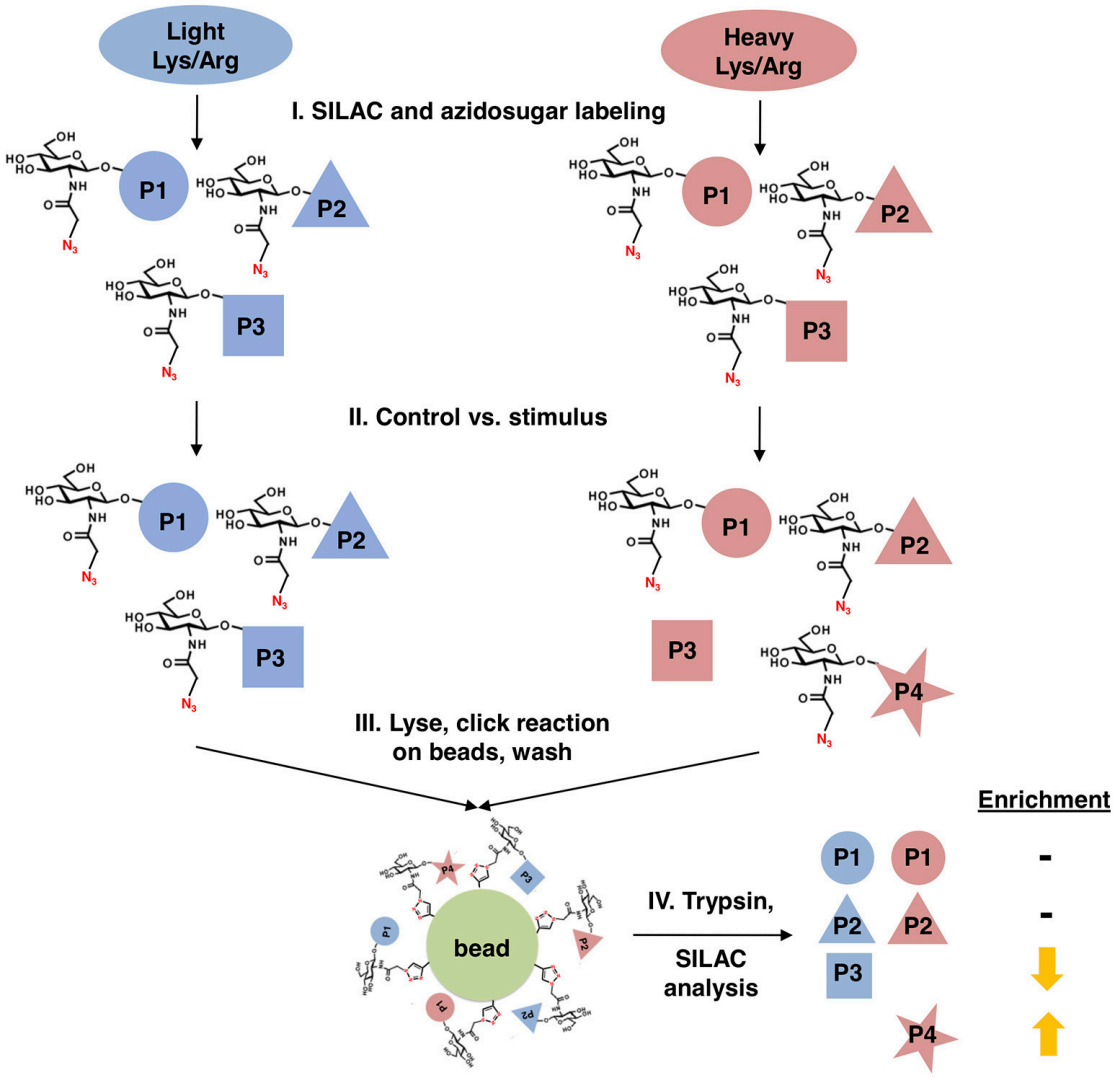
Glycoproteomics workflow. First, cells are stably labeled with either “heavy” (red) or “light” (blue) arginine and lysine to create two distinctly labeled populations, per standard SILAC protocols. Next, cells are treated with GalNAz for a brief period to allow incorporation into endogenous OGT substrates, while limiting the labeling of “housekeeping” proteins. One cell population is treated with a stimulus, inducing changes in O-GlcNAcylation, while the other remains an untreated control. Both cell populations are mixed, fractionated to remove secretory pathway glycans, and covalently ligated to an alkyne-functionalized agarose bead via a click reaction. After extremely stringent washing, the covalently captured glycoproteins are trypsinized on-bead and the resulting peptides are analyzed by quantitative SILAC MS proteomics.

We designed a new quantitative glycoproteomics strategy to discover changes in O-GlcNAcylated proteins in response to physiological stimuli, stress, or other cues. In this workflow (Figure 1), cells are first labeled with “light” ^12^C_6_^14^N_2_-lysine and ^12^${\text{C}}_{6}^{14}$N_4_-arginine or “heavy” ^13^C_6_^15^N_2_-lysine and ^13^C_6_^15^N_4_-arginine, in a standard stable isotope labeling of amino acids in cell culture (SILAC) quantitative proteomics protocol (69, 70). Next, all cells are metabolically labeled with a short pulse of GalNAz to prime the biosynthesis of UDP-GlcNAz. Then, one cell population is treated with the stimulus of interest, leaving the other as a control. All cells are then mixed, nuclear and cytoplasmic extracts are prepared by standard biochemical fractionation (to separate O-GlcNAc from secretory pathway glycans) and labeled O-GlcNAc substrates are covalently ligated to alkyne-functionalized agarose beads via a click reaction, permitting extremely stringent washing. Finally, the captured and washed glycoproteins are trypsinized on-bead, and the resulting peptides are analyzed by SILAC mass spectrometry (MS) proteomics, providing an unbiased quantitation of stimulus-dependent changes in O-GlcNAcylated proteins.

**Figure 2:**
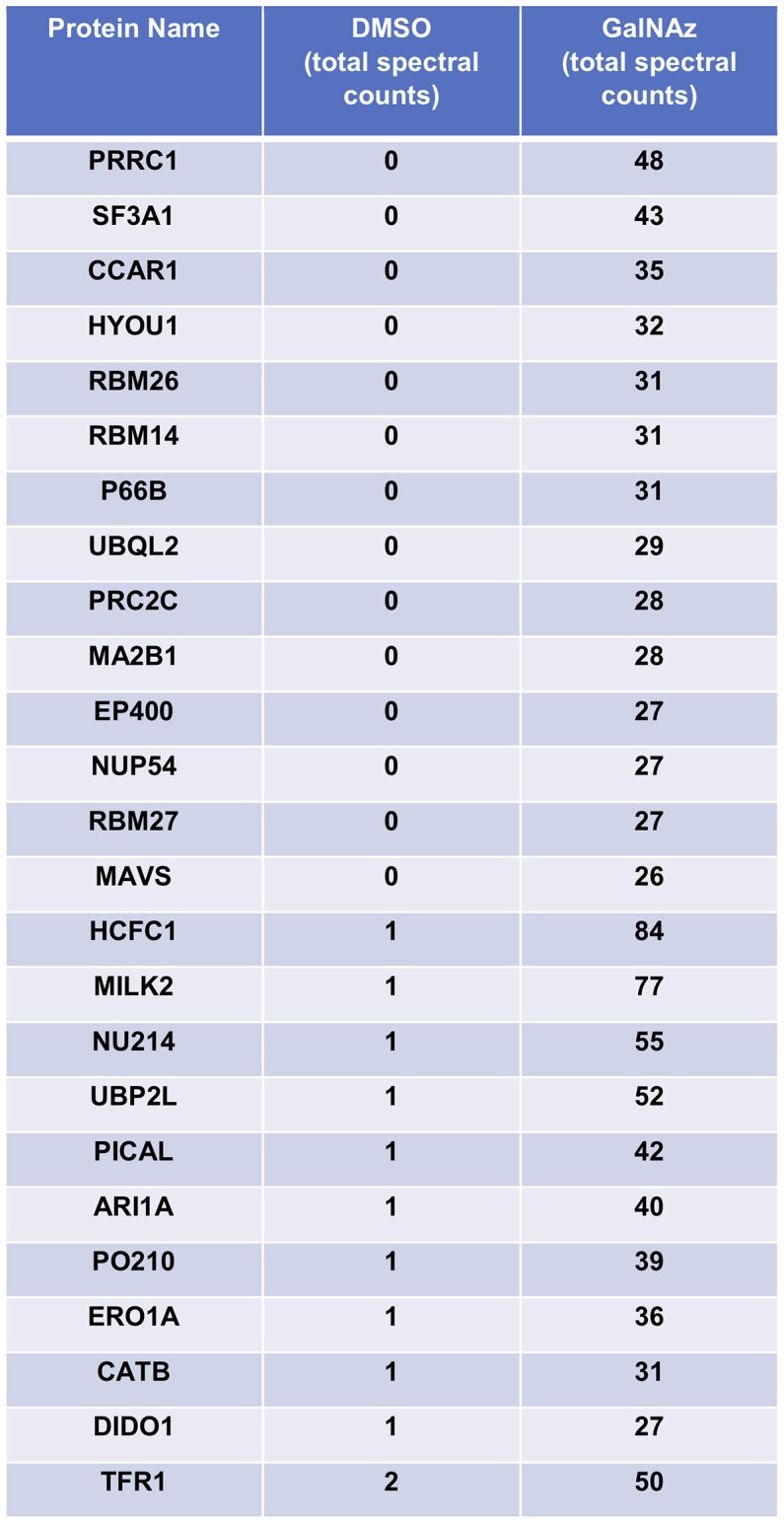
XL4.1 cells were treated with either DMSO vehicle or 100 μM GalNAz for 24 h and processed via the glycoproteomics workflow. To confirm enrichment, proteins with fewer than 25 spectral counts were excluded, and the remaining proteins were rank-ordered by the ratio of spectral counts from the vehicle and GalNAz samples (low to high). Table displays the top 25 proteins ranked this way. Commonly O-GlcNAcylated substrates (e.g., nucleoporins, HCF1) were identified exclusively in the GalNAz-treated samples, confirming the selective enrichment of endogenous OGT substrates by the workflow. Complete proteomics datasets are available as Supplemental Material.

For our pilot glycoproteomics studies, we selected the murine pro-B cell line FL5.12, subclone XL4.1 (71–73), because it is a model system for B lymphocyte activation, a process that vastly expands the protein trafficking burden through cell proliferation and augmented immunoglobulin secretion (74, 75). Previous work has also indicated that lymphocyte activation induces dramatic changes in global O-GlcNAcylation (76–78). Taken together, these reports suggested that O-GlcNAc might regulate protein trafficking in activated lymphocytes. We incubated XL4.1 cells with GalNAz or vehicle only and captured labeled proteins using our glycoproteomics workflow. Initial MS analysis revealed the strong enrichment of many known O-GlcNAcylated proteins, including numerous nucleoporins (79) and host cell factor 1 (HCF1) (80–82) (Figure 2). We concluded that our method specifically captured O-GlcNAcylated proteins, as intended.

**Figure 3:**
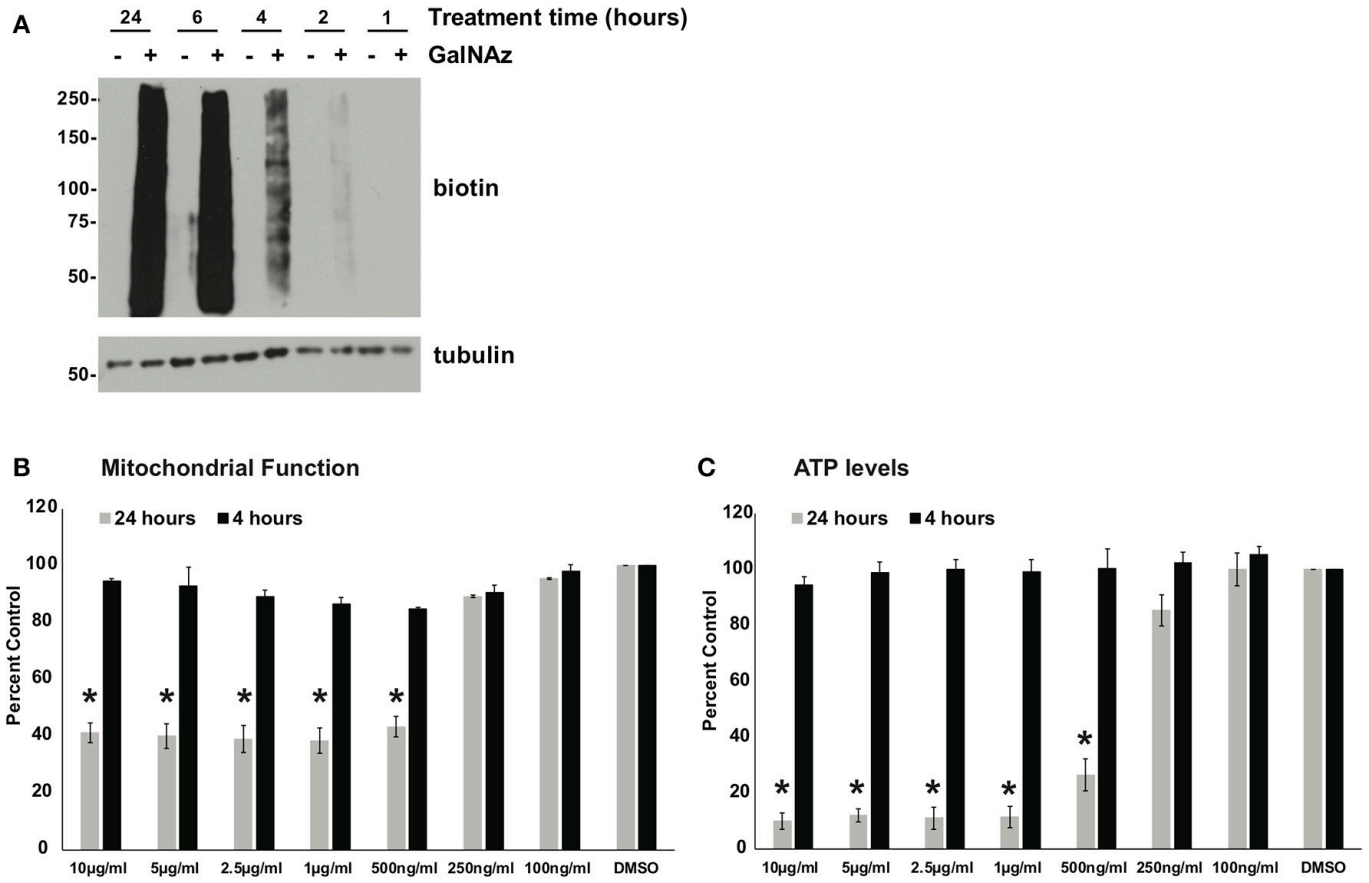
**(A)** XL4.1 cells were treated with DMSO vehicle or 100 μM GalNAz for the indicated times. Cell lysates were subjected to click reactions with an alkyne-biotin probe and analyzed by IB. GalNAz incorporation is evident as soon as 2 h after treatment. Tubulin is a loading control. **(B,C)** Parental FL5.12 N6 cells were treated with the indicated doses of BFA for 4 or 24 h and mitochondrial function **(B)** (*n* = 2) and ATP levels **(C)** (*n* = 3) were measured. Marked decreases in mitochondrial function and ATP content occurred with ≥500 ng/ml BFA treatment for 24 h, but no changes were observed at those doses after 4 h. In each assay, values were normalized to vehicle-treated control. Error bars are standard error of the mean. ^*^p < 0.05 compared to control (DMSO) by Tukey's HSD.

Next, we sought to use our glycoproteomics workflow to identify O-GlcNAcylation changes that are functionally important in protein trafficking. We reasoned that a short GalNAz pulse followed by a stimulus would afford the preferential labeling of *de novo*, stimulus-dependent changes in glycoproteins, whereas longer incubations would also label unchanging, background “housekeeping” glycoproteins, as evidenced by the enrichment of nucleoporins after long GalNAz incubation (Figure 2). We first verified that we could label endogenous XL4.1 glycoproteins with brief GalNAz incubations. We treated cells with GalNAz for various times and then reacted lysates with alkyne-biotin to label O-GlcNAc substrates. Anti-biotin immunoblot (IB) revealed that endogenous XL4.1 glycoproteins were labeled as early as 2 h after GalNAz treatment (Figure 3A). We therefore selected 2 h as a pre-stimulus GalNAz incubation time for our subsequent experiment. Next, as a model stimulus to perturb protein secretion, we selected brefeldin A (BFA), a well-characterized fungal metabolite that inhibits COPI and, secondarily, COPII vesicle trafficking (83–85). We hypothesized that secretory pathway disruption by BFA would trigger changes in O-GlcNAcylation events that regulate trafficking under homeostatic or stress conditions. To determine the lowest BFA dose that caused strong disruption of the secretory pathway, we treated apoptosis-sensitive parental FL5.12 N6 cells with a range of BFA concentrations for 4 or 24 h and measured cellular ATP and mitochondrial function (Figures 3B,C). In these experiments, 500 ng/ml was the lowest BFA dose that caused significant toxicity after 24 h while having little effect on cell viability after only 4 h (Figures 3B,C). We therefore selected 4 h of 500 ng/ml BFA as a treatment condition to disrupt protein trafficking without inducing the potentially confounding effects of downstream cell death.

**Figure 4:**
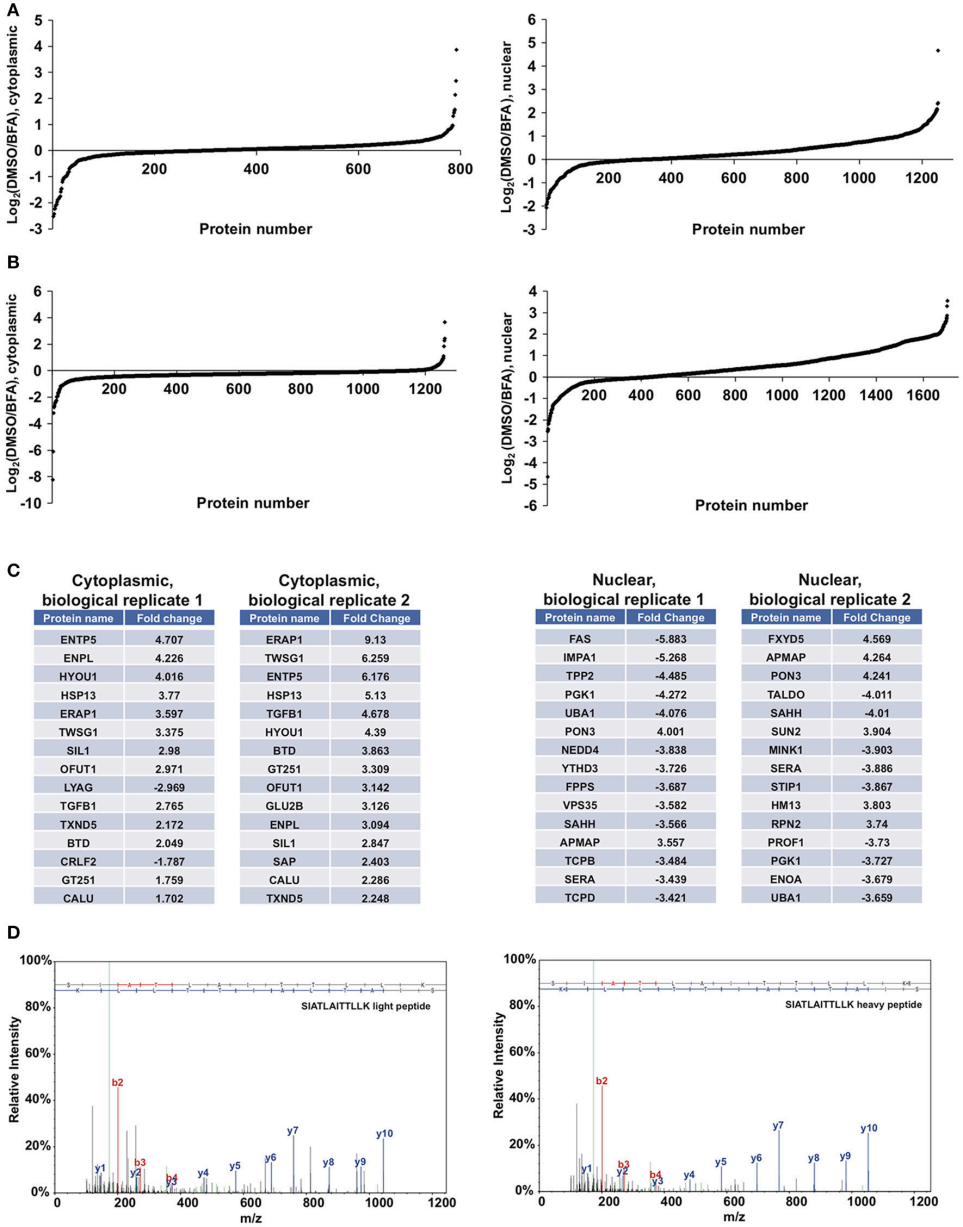
Glycoproteomics workflow detects global BFA-induced changes in O-GlcNAcylated proteins. Data for nuclear and cytoplasmic proteins are displayed as a log_2_ transform of the ratio of detected intensities from the DMSO and BFA samples, as described previously (66–68). **(A)** In the first biological replicate, 1,252 nuclear and 792 cytoplasmic proteins were identified. **(B)** In the second biological replicate, 1,703 nuclear and 1,262 cytoplasmic proteins were identified. **(C)** The nuclear and cytoplasmic proteins exhibiting concordant BFA-dependent changes (up or down) across biological replicates were rank-ordered by the magnitude of the fold-change between DMSO and BFA samples, as described previously (66–68). Tables list the top 15 nuclear and cytoplasmic proteins from each biological replicate by this ranking. Some proteins appear in both tables but at different positions, reflecting rank-order in each case. **(D)** Representative MS spectra from one SILAC biological replicate, depicting light and heavy versions of the COPγ1 peptide SIATLAITTLLK. Complete proteomics datasets are available as Supplemental Material.

We next performed proof-of-principle experiments with BFA and our glycoproteomic workflow. We treated SILAC-labeled XL4.1 cells with GalNAz for 2 h, followed by 500 ng/ml BFA (heavy-labeled cells) or vehicle control (light-labeled cells) for an additional 4 h. Then, we mixed the intact cells, derived nuclear and cytoplasmic subcellular fractions, captured GalNAz-labeled proteins and analyzed BFA-dependent changes in O-GlcNAc substrates. We calculated the fold-enrichment of every protein in our control (DMSO) vs. BFA-treated SILAC populations (1,253 nuclear and 792 cytoplasmic IDs) (Figure 4A). Overall, BFA barely altered the abundance of the vast majority of captured proteins, as expected, with 99% of both nuclear and cytoplasmic IDs changing <4-fold (Figure 4A). However, 1 nuclear and 7 cytoplasmic proteins were enriched at least 4-fold in the BFA sample vs. control, and 8 nuclear and 3 cytoplasmic proteins were depleted at least 4-fold in the BFA-treated sample. Similar results were obtained in an independent biological replicate performed with the amino acid and treatment pairings reversed (i.e., heavy/DMSO, light/BFA) (Figure 4B).

We next applied stringent filters to the data from both biological replicates to identify candidate BFA-dependent changes in O-GlcNAcylated proteins. First, we compared BFA-induced fold-changes across biological replicates and retained only protein IDs with concordant changes (up or down) across replicates. Then, we retained only nuclear proteins with a fold-change magnitude >2, and only cytoplasmic proteins with a fold-change magnitude >1.5. (A less stringent filter was placed on the cytoplasmic fraction because it exhibited fewer total protein IDs and lower-magnitude fold-changes overall). After applying these filters, we identified 80 nuclear and 17 cytoplasmic proteins displaying consistent, BFA-dependent changes across both experiments (Figure 4B). Interestingly, several of these proteins participate directly in protein trafficking, including the COPI protein COPγ1 (depleted 2.27- and 2.078-fold, respectively, from the BFA samples in the two biological replicates) and the retromer component Vps35 (depleted 3.582- and 2.412-fold from the BFA samples), or are regulators of membrane protein quality control, such as the AAA+ ATPase torsinA (enriched 2.069- and 2.466-fold in the BFA samples) and the ubiquitin E3 ligase NEDD4, which is also a known O-GlcNAc substrate (depleted 3.838- and 3.058-fold from the BFA samples) (Figures 4C,D and Supplemental Material) (86–101). We concluded that our glycoproteomics workflow identified candidate BFA-dependent changes in O-GlcNAc substrates that may impact on protein trafficking.

From our filtered glycoproteomics data, we selected COPγ1 for further validation experiments because of its well-established role in protein trafficking. COPγ1 is a core component of the heteroheptameric COPI complex, which is recruited to Golgi membranes by the small GTPase ADP ribosylation factor 1 (Arf1) to mediate vesicle formation and trafficking within the Golgi or to the ER (49, 102, 103). COPγ proteins interact with cargo adaptors in the Golgi membrane, with Arf GTPase-activating proteins and with other COPI components in the coat itself (49, 88, 89, 92). COPγ is highly conserved across eukaryotes and is essential for *in vitro* COPI vesicle formation and for viability in budding yeast (49, 104, 105). While phosphorylation, arginine methylation, and ubiquitination of COPγ1 have been observed in several studies (106–116), O-GlcNAcylation of COPγ has never been reported.

**Figure 5:**
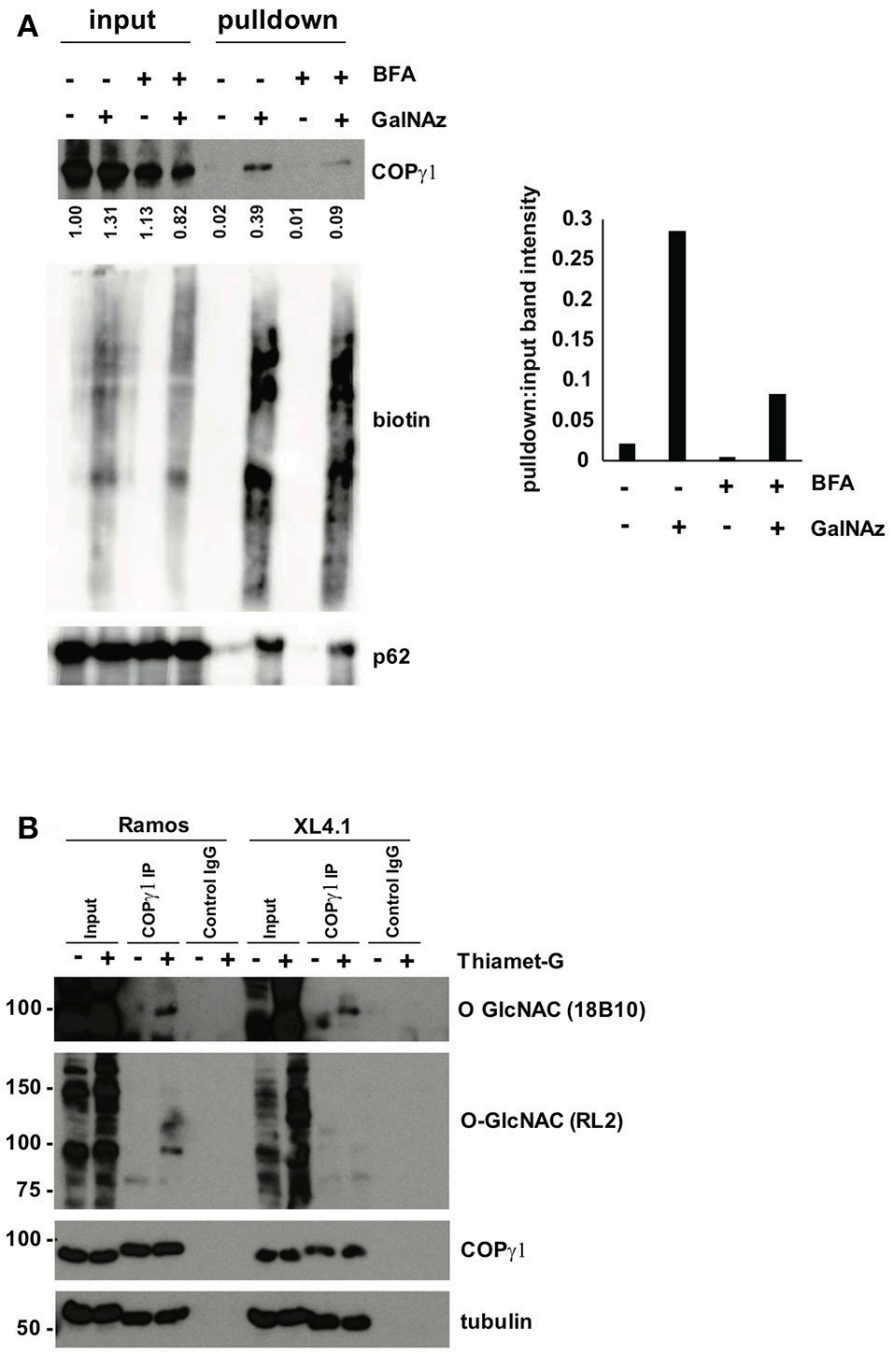
**(A)** Ramos cells were treated with 100 μM GalNAz or DMSO vehicle for 6 h, followed by 500 ng/ml BFA or DMSO vehicle for an additional 4 h, and then harvested. Nuclear and cytoplasmic fractions were prepared, subjected to click reactions with an alkyne-biotin probe and incubated with streptavidin beads for enrichment. Beads were washed and eluted proteins were analyzed by IB. Nuclear fraction IBs from a representative experiment are shown. Nucleoporin-62, a heavily O-GlcNAcylated protein, is a control for equal loading (input lanes) and biotin enrichment (pulldown lanes). Band intensities in the COPγ1 IB were quantified in ImageJ, normalized to control (DMSO, input lane) and listed below. The ratio of pulldown:input for each treatment was calculated as a measure of COPγ1GalNAz modification and is given in the graph at the right. Consistent with the glycoproteomics results, COPγ1 levels were reduced after BFA treatment, indicating a reduction in O-GlcNAcylation. Similar results were obtained with XL4.1 cells (not shown). (**B**) Ramos (left) and XL4.1 (right) cells were treated with 50 μM Thiamet-G or DMSO vehicle for 8 h and lysates were subjected to anti-COPγ1IP and analyzed via IB.

To confirm our MS results with COPγ1, we GalNAz-labeled XL4.1 or Ramos cells (a human B cell line) in the presence or absence of BFA treatment, performed click reactions with alkyne-biotin, purified O-GlcNAc substrates by streptavidin affinity chromatography and analyzed the results by IB (Figure 5A). Consistent with our glycoproteomics results, anti-COPγ1 IB indicated that BFA treatment reduced the O-GlcNAcylation of COPγ1 without causing dramatic effects on total COPγ1 levels (Figure 5A). To extend these results to natural O-GlcNAc, we immunoprecipitated (IP-ed) endogenous COPγ1 from XL4.1 or Ramos cells and observed that it was recognized by anti-O-GlcNAc monoclonal antibodies (Figure 5B). This signal was specific, because treatment of cells with Thiamet-G, a small molecule inhibitor of OGA (64), increased both global O-GlcNAc signal and anti-O-GlcNAc immunoreactivity of COPγ1 (Figure 5B). We concluded that endogenous COPγ1 is dynamically O-GlcNAcylated in mammalian cells under homeostatic conditions, and deglycosylated upon disruption of protein trafficking by BFA.

**Figure 6:**
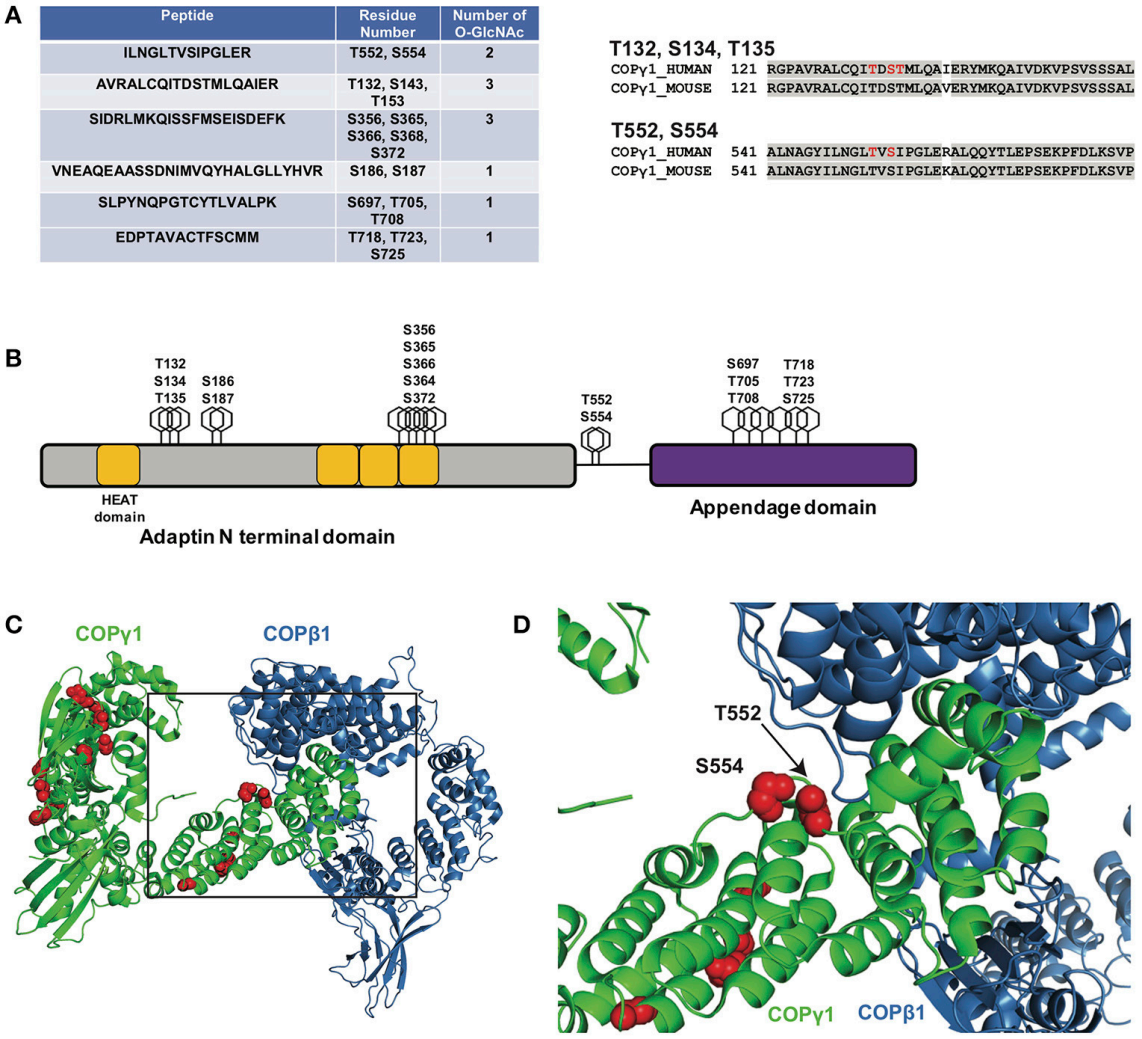
(**A**, left) Myc-6xHis-tagged human COPγ1 was tandem-purified to homogeneity from transfected 293T cells and analyzed via ETD/HCD-MS. Peptide sequence, number of O-GlcNAc moieties detected, and number of serine and threonine residues are displayed for tryptic peptides exhibiting glycosylation. (**A**, right) Alignment of human and mouse COPγ1 regions with unambiguously assigned O-GlcNAc modification sites, denoted in red. Human and mouse COPγ1 are 97.3% identical and are 99.4% similar. All O-GlcNAc sites identified on human COPγ1 are conserved in the mouse ortholog. **(B)** Schematic depicting candidate O-GlcNAc sites on the COPγ1 domain structure. The C-terminal appendage domain contains an ARFGAP2-interacting region and binds the α/β/ε COPI subcomplex. **(C)** Interface of COPγ1 (green) and COPβ1 (blue), with potential O-GlcNAc sites in red, from the previously reported structure of the COPI triad (PDB: 5A1U) (117). **(D)** Zoomed view from **(C)** (black box) of the COPγ1/COPβ1 interface. T552 and S554, two unambiguously assigned O-GlcNAc sites on COPγ1, lie in close proximity to COPβ1. Complete proteomics datasets are available as Supplemental Material.

As a first step toward characterizing the function of COPγ1 O-GlcNAcylation, we expressed and purified epitope-tagged human COPγ1 to homogeneity from human cells and used MS to map O-GlcNAc-modified residues. We detected 11 O-GlcNAc moieties across six unique peptides and unambiguously assigned five glycosylation sites: T132, S134, T135, T552, and S554 (Figure 6A and Supplemental Material). COPγ1 is highly conserved between human and mouse (97.3% identical and 99.4% similar), and all candidate O-GlcNAc sites that we identified are identical between the orthologs (Figures 6A,B). The candidate O-GlcNAc sites occur in several regions of the COPγ1 protein, with most lying within the last HEAT repeat of the adaptin N-terminal domain or in the appendage domain (Figure 6B). The appendage domain interacts with ARFGAP2, which binds the α/β/ε COPI subcomplex and influences vesicle uncoating, suggesting that O-GlcNAcylation in this domain could influence these functions (88). Finally, we modeled the observed O-GlcNAc sites onto crystal structures of COPγ1 in the COPI coat “triad” complex (Figures 6C,D) (PDB: 5A1U) (117). Interestingly, the T552 and S554 glycosylation sites of COPγ1 lie close to COPβ1-binding interface and might impact on this interaction, which is essential for COPI function (117). Taken together, our BFA and MS results suggest that site-specific COPγ1 O-GlcNAcylation may promote or license its activity in the COPI pathway.

## Discussion

O-GlcNAc is a highly dynamic PTM that modifies thousands of nuclear, cytoplasmic and mitochondrial proteins. While the number of identified O-GlcNAc substrates continues to rise, the specific functions of O-GlcNAc on most proteins are elusive and assessing stimulus-triggered changes in O-GlcNAcylated proteins remains a significant challenge in the field. Here, we report a novel glycoproteomics workflow enabling the proteome-wide identification and quantification of changes in O-GlcNAc-modified proteins and use it to discover cycling O-GlcNAcylation of mammalian COPγ1 as a candidate regulatory event in Golgi protein trafficking.

Several proteomics-compatible approaches for enriching natural O-GlcNAc exist, including lectin weak-affinity chromatography (LWAC), chemical modification of O-GlcNAc moieties (e.g., β-elimination followed by Michael addition) and chemoenzymatic methods that harness an engineered galactosyltransferase (9, 59, 81, 82, 118–134). Each of these is a well-established and powerful tool for elucidating O-GlcNAc signaling. Our glycoproteomics workflow leverages GalNAz metabolic labeling, which complements these methods in important ways. In our approach, a pulse of GalNAz is added to cultured cells only shortly before the stimulus of interest, permitting the preferential enrichment and characterization of relatively new glycosylation changes. Therefore, our workflow provides time resolution and reduces the labeling of long-lived, unchanging O-GlcNAc moieties (e.g., on the nuclear pore complex) that could otherwise dominate the proteomics results (Figure 2). In addition, our workflow affords the covalent capture of O-GlcNAcylated proteins onto a solid matrix, allowing extremely stringent washing conditions to remove unglycosylated proteins. Although the individual components of our strategy have been reported previously, we have assembled them into a new and optimized workflow in which the proteome-wide profiling of stimulus-dependent O-GlcNAc changes in SILAC-labeled cells can be performed by one worker in as little as 1 week. Moreover, our workflow can be implemented in any cell type or organism that supports azidosugar and SILAC labeling, and can be used to study a wide range of stimuli, stresses or other experimental comparisons. We anticipate that this method will be a useful addition to the quantitative analysis of O-GlcNAc signaling.

In pilot experiments, we used our workflow to address the role of O-GlcNAcylation changes in mammalian protein trafficking, using BFA as an established tool compound. These studies identified the dynamic glycosylation of COPγ1 in mammalian cells (Figures 4–6 and Supplemental Material), and validation experiments demonstrated that endogenous COPγ1 is reversibly modified by natural O-GlcNAc atleast 11 sites, confirming the utility of our approach in characterizing native signaling pathways (Figures 5, 6). (We note that numerous Golgi proteins partitioned to the “nuclear” sample in our fractionation procedure, likely explaining the presence of COPγ1—see full proteomics datasets in the Supplemental Material).

COPI trafficking relies on the guanine nucleotide exchange factor GBP1 to exchange GDP for GTP on Arf1 (47–49, 102, 103). Arf1 undergoes a conformational change upon GTP binding, inserting an N-terminal amphipathic α-helix into the Golgi membrane (49, 102, 103). Membrane-bound Arf1 then recruits the stable heteroheptameric COPI coat complex, which includes COPγ1 (49, 102, 103, 135). The assembling COPI heteroheptamers also undergo a major conformational change, promoting oligomerization of the coat complex and eventual vesicle formation and scission (117, 135, 136). BFA perturbs protein trafficking by stabilizing an abortive intermediate of the Arf1 complex, disrupting both the COPI and, subsequently, the COPII pathways (83–85).

Our results suggest that COPγ1 glycosylation may regulate protein trafficking within or from the Golgi. Consistent with this hypothesis, a prior proteomics study identified a putative biochemical interaction between OGT and the COPI component COPε (137). The authors proposed that O-GlcNAc might govern intra-Golgi vesicle transport, although no direct glycosylation of any COPI protein was demonstrated (137). The precise biochemical and functional effects of O-GlcNAcylation on COPγ1 remain to be determined, but our results, combined with prior reports, suggest several possibilities. First, because O-GlcNAc can regulate protein-protein interactions in a variety of contexts (138), glycosylation may affect the interaction of COPγ1 with specific binding partners, such as COPβ1, COPζ, Arf1, or p24 cargo adaptors (49, 105, 139–143). Consistent with this notion, our MS site-mapping revealed O-GlcNAcylation on two sites, T552 and S554, located in close proximity to the interface with COPβ1 in the COPI triad structure (Figures 6C,D) (117). Addition of one or more bulky O-GlcNAc moieties in this region of COPγ1 may alter this interaction, which is essential for COPI function. Second, O-GlcNAcylation of COPγ1 may promote or inhibit one of the significant conformational changes that occur during COPI coat assembly (49, 102, 103, 117, 135, 136, 142, 143). Third, O-GlcNAcylation of COPγ1 may regulate the membrane recruitment of the heteroheptameric complex. We have previously demonstrated an analogous role for O-GlcNAc signaling in the COPII pathway, as OGA inhibition impairs the membrane recruitment of the COPII proteins Sec31A and Sec23A (54). Fourth, O-GlcNAc may regulate COPγ1 through cross-talk with other PTMs. Interestingly, five of the candidate O-GlcNAc sites we identified on COPγ1 (S356, S554, T718, T723, and S725) are also reported phosphorylation sites (144). Therefore, COPγ1 function may be regulated by the well-documented, complex interplay between O-GlcNAcylation and O-phosphorylation (2, 145–149). Our site-mapping data have paved the way for future studies to test these hypotheses, and experiments with single and compound glycosylation site mutants are currently underway to determine the impact of COPγ1 O-GlcNAcylation in live-cell trafficking assays.

While many excellent studies have dissected the structures and functions of the core COPI machinery (49, 142, 143), much less is known about how this critical pathway is regulated by mammalian cells in response to rapidly changing physiological and pathological signals. PTMs likely serve as one important mode of COPI regulation. Indeed, several proteomics studies have reported phosphorylation, ubiquitination and arginine methylation of COPγ in particular (107–116), and one study provided functional evidence that phosphorylation of COPβ and COPγ influences coatomer assembly or membrane recruitment (106). Therefore, COPI trafficking may be governed in part by COPγ PTMs. Our results indicate that O-GlcNAc may be a functionally important PTM in the COPI system as well. Moreover, we detected putative BFA-dependent O-GlcNAc changes on proteins operating in distinct parts of the secretory pathway, including Vps35, torsinA, and NEDD4 (Figure 4), and previous studies have implicated O-GlcNAcylation in other vesicle transport pathways beyond COPI as well (28, 50–63). Based on these observations, we propose that O-GlcNAcylation may be a widespread mode of dynamic regulation in mammalian protein trafficking.

## Data availability statement

All datasets generated in this study are included in the manuscript and supplementary files.

## Author contributions

NC, ES, and MB contributed conception and design of the study. NC, PL, and TS performed experiments and prepared samples. NC and ES performed and analyzed glycoproteomics experiments. BB performed statistical analysis of cell viability data and analyses of O-GlcNAc site-mapping data in the context of previously reported COPγ1 structures. NC and MB wrote the manuscript, and all authors contributed to manuscript revision and read and approved the submitted version.

### Conflict of interest statement

The authors declare that the research was conducted in the absence of any commercial or financial relationships that could be construed as a potential conflict of interest.

## References

[1] Hanover JA Krause MW Love DC The hexosamine signaling pathway: O-GlcNAc cycling in feast or famine. Biochim Biophys Acta (2010) 1800:80–95. 10.1016/j.bbagen.2009.07.017 19647043 PMC2815088

[2] Hart GW Slawson C Ramirez-Correa G Lagerlof O. Cross talk between O-GlcNAcylation and phosphorylation: roles in signaling, transcription, and chronic disease. Annu Rev Biochem. (2011) 80:825–58. 10.1146/annurev-biochem-060608-102511 21391816 PMC3294376

[3] Hart GW. Three decades of research on O-GlcNAcylation - a major nutrient sensor that regulates signaling, transcription and cellular metabolism. Front Endocrinol. (2014) 5:183. 10.3389/fendo.2014.00183 25386167 PMC4209869

[4] Bond MR Hanover JA. A little sugar goes a long way: the cell biology of O-GlcNAc. J Cell Biol. (2015) 208:869–80. 10.1083/jcb.201501101 25825515 PMC4384737

[5] Lefebvre T Issad T. 30 Years Old: O-GlcNAc reaches the age of reason - regulation of cell signaling and metabolism by O-GlcNAcylation. Front Endocrinol. (2015) 6:17. 10.3389/fendo.2015.00017 25709599 PMC4321574

[6] Levine ZG Walker S. The biochemistry of O-GlcNAc transferase: which functions make it essential in mammalian cells? Annu Rev Biochem. (2016) 85:631–57. 10.1146/annurev-biochem-060713-035344 27294441

[7] Yang X Qian K. Protein O-GlcNAcylation: emerging mechanisms and functions. Nat Rev Mol Cell Biol. (2017) 18:452–65. 10.1038/nrm.2017.22 28488703 PMC5667541

[8] Dehennaut V Leprince D Lefebvre T. O-GlcNAcylation, an epigenetic mark. focus on the histone code, TET family proteins, and polycomb group proteins. Front Endocrinol. (2014) 5:155. 10.3389/fendo.2014.00155 25309514 PMC4176146

[9] Yi W Clark PM Mason DE Keenan MC Hill C Goddard WA III. Phosphofructokinase 1 glycosylation regulates cell growth and metabolism. Science (2012) 337:975–80. 10.1126/science.1222278 22923583 PMC3534962

[10] Ma Z Vosseller K. O-GlcNAc in cancer biology. Amino Acids (2013) 45:719–33. 10.1007/s00726-013-1543-8 23836420

[11] Singh JP Zhang K Wu J Yang X. O-GlcNAc signaling in cancer metabolism and epigenetics. Cancer Lett. (2015) 356(2 Pt A):244–50. 10.1016/j.canlet.2014.04.014 24769077 PMC4208982

[12] Ferrer CM Sodi VL Reginato MJ. O-GlcNAcylation in cancer biology: linking metabolism and signaling. J Mol Biol. (2016) 428:3282–94. 10.1016/j.jmb.2016.05.028 27343361 PMC4983259

[13] Ma J Hart GW. Protein O-GlcNAcylation in diabetes and diabetic complications. Expert Rev Proteomics (2013) 10:365–80. 10.1586/14789450.2013.820536 23992419 PMC3985334

[14] Baudoin L Issad T. O-GlcNAcylation and inflammation: a vast territory to explore. Front Endocrinol. (2014) 5:235. 10.3389/fendo.2014.00235 25620956 PMC4288382

[15] Hardiville S Hart GW. Nutrient regulation of signaling, transcription, and cell physiology by O-GlcNAcylation. Cell Metab. (2014) 20:208–13. 10.1016/j.cmet.2014.07.014 25100062 PMC4159757

[16] Vaidyanathan K Wells L. Multiple tissue-specific roles for the O-GlcNAc post-translational modification in the induction of and complications arising from type II diabetes. J Biol Chem. (2014) 289:34466–71. 10.1074/jbc.R114.591560 25336652 PMC4263854

[17] Darley-Usmar VM Ball LE Chatham JC. Protein O-linked beta-N-acetylglucosamine: a novel effector of cardiomyocyte metabolism and function. J Mol Cell Cardiol. (2012) 52:538–49. 10.1016/j.yjmcc.2011.08.009 21878340 PMC3928598

[18] Erickson JR Pereira L Wang L Han G Ferguson A Dao K. Diabetic hyperglycaemia activates CaMKII and arrhythmias by O-linked glycosylation. Nature (2013) 502:372–6. 10.1038/nature12537 24077098 PMC3801227

[19] Dassanayaka S Jones SP. O-GlcNAc and the cardiovascular system. Pharmacol Ther. (2014) 142:62–71. 10.1016/j.pharmthera.2013.11.005 24287310 PMC3943723

[20] Erickson JR. Mechanisms of CaMKII activation in the heart. Front Pharmacol. (2014) 5:59. 10.3389/fphar.2014.00059 24765077 PMC3980116

[21] Yuzwa SA Shan X Macauley MS Clark T Skorobogatko Y Vosseller K. Increasing O-GlcNAc slows neurodegeneration and stabilizes tau against aggregation. Nat Chem Biol. (2012) 8:393–9. 10.1038/nchembio.797 22366723

[22] Vaidyanathan K Durning S Wells L. Functional O-GlcNAc modifications: implications in molecular regulation and pathophysiology. Crit Rev Biochem Mol Biol. (2014) 49:140–63. 10.3109/10409238.2014.884535 24524620 PMC4912837

[23] Yuzwa SA Vocadlo DJ. O-GlcNAc and neurodegeneration: biochemical mechanisms and potential roles in Alzheimer's disease and beyond. Chem Soc Rev. (2014) 43:6839–58. 10.1039/C4CS00038B 24759912

[24] Zhu Y Shan X Yuzwa SA Vocadlo DJ. The emerging link between O-GlcNAc and Alzheimer disease. J Biol Chem. (2014) 289:34472–81. 10.1074/jbc.R114.601351 25336656 PMC4263855

[25] Mondoux MA Love DC Ghosh SK Fukushige T Bond M Weerasinghe GR. O-linked-N-acetylglucosamine cycling and insulin signaling are required for the glucose stress response in Caenorhabditis elegans. Genetics (2011) 188:369–82. 10.1534/genetics.111.126490 21441213 PMC3122314

[26] Bond MR Hanover JA. O-GlcNAc cycling: a link between metabolism and chronic disease. Annu Rev Nutr. (2013) 33:205–29. 10.1146/annurev-nutr-071812-161240 23642195 PMC10483992

[27] Boyce M Bertozzi CR. Bringing chemistry to life. Nat Methods (2011) 8:638–42. 10.1038/nmeth.1657 21799498 PMC3184769

[28] Boyce M Carrico IS Ganguli AS Yu SH Hangauer MJ Hubbard SC. Metabolic cross-talk allows labeling of O-linked beta-N-acetylglucosamine-modified proteins via the N-acetylgalactosamine salvage pathway. Proc Natl Acad Sci USA. (2011) 108:3141–6. 10.1073/pnas.1010045108 21300897 PMC3044403

[29] Palaniappan KK Hangauer MJ Smith TJ Smart BP Pitcher AA Cheng EH. A chemical glycoproteomics platform reveals O-GlcNAcylation of mitochondrial voltage-dependent anion channel 2. Cell Rep. (2013) 5:546–52. 10.1016/j.celrep.2013.08.048 24120863 PMC3869705

[30] Chen PH Smith TJ Wu J Siesser PF Bisnett BJ Khan F. Glycosylation of KEAP1 links nutrient sensing to redox stress signaling. EMBO J. (2017) 36:2233–50. 10.15252/embj.201696113 28663241 PMC5538768

[31] Tarbet HJ Dolat L Smith TJ Condon BM O'Brien ET III Valdivia RH. Site-specific glycosylation regulates the form and function of the intermediate filament cytoskeleton. Elife (2018) 7:e31807. 10.7554/eLife.31807 29513221 PMC5841932

[32] Hang HC Yu C Kato DL Bertozzi CR. A metabolic labeling approach toward proteomic analysis of mucin-type O-linked glycosylation. Proc Natl Acad Sci USA. (2003) 100:14846–51. 10.1073/pnas.2335201100 14657396 PMC299823

[33] Rostovtsev VV Green LG Fokin VV Sharpless KB. A stepwise huisgen cycloaddition process: copper(I)-catalyzed regioselective “ligation” of azides and terminal alkynes. Angew Chem Int Ed Engl. (2002) 41:2596–9. 10.1002/1521-3773(20020715)41:14<2596::AID-ANIE2596>3.0.CO;2-4 12203546

[34] Tornoe CW Christensen C Meldal M. Peptidotriazoles on solid phase: [1,2,3]-triazoles by regiospecific copper(i)-catalyzed 1,3-dipolar cycloadditions of terminal alkynes to azides. J Org Chem. (2002) 67:3057–64. 10.1021/jo011148j 11975567

[35] McKay CS Finn MG. Click chemistry in complex mixtures: bioorthogonal bioconjugation. Chem Biol. (2014) 21:1075–101. 10.1016/j.chembiol.2014.09.002 25237856 PMC4331201

[36] Li L Zhang Z. Development and applications of the copper-catalyzed Azide-Alkyne Cycloaddition (CuAAC) as a Bioorthogonal Reaction. Molecules (2016) 21:E1393. 10.3390/molecules21101393 27783053 PMC6273301

[37] Kaufman RJ. Stress signaling from the lumen of the endoplasmic reticulum: coordination of gene transcriptional and translational controls. Genes Dev. (1999) 13:1211–33. 10.1101/gad.13.10.1211 10346810

[38] Huh WK Falvo JV Gerke LC Carroll AS Howson RW Weissman JS. Global analysis of protein localization in budding yeast. Nature (2003) 425:686–91. 10.1038/nature02026 14562095

[39] Barlowe CK Miller EA. Secretory protein biogenesis and traffic in the early secretory pathway. Genetics (2013) 193:383–410. 10.1534/genetics.112.142810 23396477 PMC3567731

[40] Mettlen M Chen PH Srinivasan S Danuser G Schmid SL. Regulation of clathrin-mediated endocytosis. Annu Rev Biochem. (2018) 87:871–96. 10.1146/annurev-biochem-062917-012644 29661000 PMC6383209

[41] Baker D Hicke L Rexach M Schleyer M Schekman R. Reconstitution of SEC gene product-dependent intercompartmental protein transport. Cell (1988) 54:335–44. 10.1016/0092-8674(88)90196-1 3293799

[42] Ruohola H Kabcenell AK Ferro-Novick S. Reconstitution of protein transport from the endoplasmic reticulum to the Golgi complex in yeast: the acceptor Golgi compartment is defective in the sec23 mutant. J Cell Biol. (1988) 107:1465–76. 10.1083/jcb.107.4.1465 3049622 PMC2115264

[43] Barlowe C Orci L Yeung T Hosobuchi M Hamamoto S Salama N. COPII: a membrane coat formed by Sec proteins that drive vesicle budding from the endoplasmic reticulum. Cell (1994) 77:895–907. 10.1016/0092-8674(94)90138-4 8004676

[44] Routledge KE Gupta V Balch WE. Emergent properties of proteostasis-COPII coupled systems in human health and disease. Mol Membr Biol. (2010) 27:385–97. 10.3109/09687688.2010.524894 21054154

[45] Brandizzi F Barlowe C. Organization of the ER-Golgi interface for membrane traffic control. Nat Rev Mol Cell Biol. (2013) 14:382–92. 10.1038/nrm3588 23698585 PMC4064004

[46] Miller EA Schekman R. COPII - a flexible vesicle formation system. Curr Opin Cell Biol. (2013) 25:420–7. 10.1016/j.ceb.2013.04.005 23702145 PMC3736695

[47] Guo Y Sirkis DW Schekman R. Protein sorting at the trans-Golgi network. Annu Rev Cell Dev Biol. (2014) 30:169–206. 10.1146/annurev-cellbio-100913-013012 25150009

[48] Rout MP Field MC. The evolution of organellar coat complexes and organization of the eukaryotic cell. Annu Rev Biochem. (2017) 86:637–57. 10.1146/annurev-biochem-061516-044643 28471691

[49] Bethune J Wieland FT. Assembly of COPI and COPII vesicular coat proteins on membranes. Annu Rev Biophys. (2018) 47:63–83. 10.1146/annurev-biophys-070317-033259 29345989

[50] Dudognon P Maeder-Garavaglia C Carpentier JL Paccaud JP. Regulation of a COPII component by cytosolic O-glycosylation during mitosis. FEBS Lett. (2004) 561:44–50. 10.1016/S0014-5793(04)00109-7 15013749

[51] Teo CF Ingale S Wolfert MA Elsayed GA Not LG Chatham JC. Glycopeptide-specific monoclonal antibodies suggest new roles for O-GlcNAc. Nat Chem Biol. (2010) 6:338–43. 10.1038/nchembio.338 20305658 PMC2857662

[52] Zachara NE Molina H Wong KY Pandey A Hart GW. The dynamic stress-induced “O-GlcNAc-ome” highlights functions for O-GlcNAc in regulating DNA damage/repair and other cellular pathways. Amino Acids (2011) 40:793–808. 10.1007/s00726-010-0695-z 20676906 PMC3329784

[53] Lee A Miller D Henry R Paruchuri VD O'Meally RN Boronina T. Combined antibody/lectin enrichment identifies extensive changes in the O-GlcNAc Sub-proteome upon oxidative stress. J Proteome Res. (2016) 15:4318–36. 10.1021/acs.jproteome.6b00369 27669760 PMC8132933

[54] Cox NJ Unlu G Bisnett BJ Meister TR Condon BM Luo PM. Dynamic glycosylation governs the vertebrate COPII protein trafficking pathway. Biochemistry (2018) 57:91–107. 10.1021/acs.biochem.7b00870 29161034 PMC5767944

[55] Murphy JE Hanover JA Froehlich M DuBois G Keen JH. Clathrin assembly protein AP-3 is phosphorylated and glycosylated on the 50-kDa structural domain. J Biol Chem. (1994) 269:21346–52. 8063760

[56] Yao PJ Coleman PD. Reduced O-glycosylated clathrin assembly protein AP180: implication for synaptic vesicle recycling dysfunction in Alzheimer's disease. Neurosci Lett. (1998) 252:33–6. 10.1016/S0304-3940(98)00547-3 9756352

[57] Yao PJ Coleman PD. Reduction of O-linked N-acetylglucosamine-modified assembly protein-3 in Alzheimer's disease. J Neurosci. (1998) 18:2399–411. 10.1523/JNEUROSCI.18-07-02399.1998 9502801 PMC6793091

[58] Akimoto Y Comer FI Cole RN Kudo A Kawakami H Hirano H. Localization of the O-GlcNAc transferase and O-GlcNAc-modified proteins in rat cerebellar cortex. Brain Res. (2003) 966:194–205. 10.1016/S0006-8993(02)04158-6 12618343

[59] Vosseller K Trinidad JC Chalkley RJ Specht CG Thalhammer A Lynn AJ. O-Linked N-acetylglucosamine proteomics of postsynaptic density preparations using lectin weak affinity chromatography and mass spectrometry. Mol Cell Proteomics (2006) 5:923–34. 10.1074/mcp.T500040-MCP200 16452088

[60] Tallent MK Varghis N Skorobogatko Y Hernandez-Cuebas L Whelan K Vocadlo DJ. *In vivo* modulation of O-GlcNAc levels regulates hippocampal synaptic plasticity through interplay with phosphorylation. J Biol Chem. (2009) 284:174–81. 10.1074/jbc.M807431200 19004831

[61] Graham ME Thaysen-Andersen M Bache N Craft GE Larsen MR Packer NH. A novel post-translational modification in nerve terminals: O-linked N-acetylglucosamine phosphorylation. J Proteome Res. (2011) 10:2725–33. 10.1021/pr1011153 21500857

[62] Chun YS Park Y Oh HG Kim TW Yang HO Park MK. O-GlcNAcylation promotes non-amyloidogenic processing of amyloid-beta protein precursor via inhibition of endocytosis from the plasma membrane. J Alzheimers Dis. (2015) 44:261–75. 10.3233/JAD-140096 25208619

[63] Chun YS Kwon OH Chung S. O-GlcNAcylation of amyloid-beta precursor protein at threonine 576 residue regulates trafficking and processing. Biochem Biophys Res Commun. (2017) 490:486–91. 10.1016/j.bbrc.2017.06.067 28624365

[64] Yuzwa SA Macauley MS Heinonen JE Shan X Dennis RJ He Y. A potent mechanism-inspired O-GlcNAcase inhibitor that blocks phosphorylation of tau *in vivo*. Nat Chem Biol. (2008) 4:483–90. 10.1038/nchembio.96 18587388

[65] Lossner C Warnken U Pscherer A Schnolzer M. Preventing arginine-to-proline conversion in a cell-line-independent manner during cell cultivation under stable isotope labeling by amino acids in cell culture (SILAC) conditions. Anal Biochem. (2011) 412:123–5. 10.1016/j.ab.2011.01.011 21241653

[66] Foster MW Yang Z Gooden DM Thompson JW Ball CH Turner ME. Proteomic characterization of the cellular response to nitrosative stress mediated by s-nitrosoglutathione reductase inhibition. J Proteome Res. (2012) 11:2480–91. 10.1021/pr201180m 22390303 PMC3327136

[67] Yang W Thompson JW Wang Z Wang L Sheng H Foster MW. Analysis of oxygen/glucose-deprivation-induced changes in SUMO3 conjugation using SILAC-based quantitative proteomics. J Proteome Res. (2012) 11:1108–17. 10.1021/pr200834f 22082260 PMC3628696

[68] Foster MW Thompson JW Forrester MT Sha Y McMahon TJ Bowles DE. Proteomic analysis of the NOS2 interactome in human airway epithelial cells. Nitric Oxide (2013) 34:37–46. 10.1016/j.niox.2013.02.079 23438482 PMC3769490

[69] Ong SE Blagoev B Kratchmarova I Kristensen DB Steen H Pandey A. Stable isotope labeling by amino acids in cell culture, SILAC, as a simple and accurate approach to expression proteomics. Mol Cell Proteomics (2002) 1:376–86. 10.1074/mcp.M200025-MCP200 12118079

[70] Terzi F Cambridge S. An overview of advanced SILAC-labeling strategies for quantitative proteomics. Methods Enzymol. (2017) 585:29–47. 10.1016/bs.mie.2016.09.014 28109435

[71] Rathmell JC Vander Heiden MG Harris MH Frauwirth KA Thompson CB In the absence of extrinsic signals, nutrient utilization by lymphocytes is insufficient to maintain either cell size or viability. Mol Cell (2000) 6:683–92. 10.1016/S1097-2765(00)00066-6 11030347

[72] Wieman HL Wofford JA Rathmell JC. Cytokine stimulation promotes glucose uptake via phosphatidylinositol-3 kinase/Akt regulation of Glut1 activity and trafficking. Mol Biol Cell (2007) 18:1437–46. 10.1091/mbc.e06-07-0593 17301289 PMC1838986

[73] Caro-Maldonado A Gerriets VA Rathmell JC. Matched and mismatched metabolic fuels in lymphocyte function. Semin Immunol. (2012) 24:405–13. 10.1016/j.smim.2012.12.002 23290889 PMC3582857

[74] Shaffer AL Shapiro-Shelef M Iwakoshi NN Lee AH Qian SB Zhao H. XBP1, downstream of Blimp-1, expands the secretory apparatus and other organelles, and increases protein synthesis in plasma cell differentiation. Immunity (2004) 21:81–93. 10.1016/j.immuni.2004.06.010 15345222

[75] Ron D Walter P. Signal integration in the endoplasmic reticulum unfolded protein response. Nat Rev Mol Cell Biol. (2007) 8:519–29. 10.1038/nrm2199 17565364

[76] Kearse KP Hart GW. Lymphocyte activation induces rapid changes in nuclear and cytoplasmic glycoproteins. Proc Natl Acad Sci USA. (1991) 88:1701–5. 10.1073/pnas.88.5.1701 2000378 PMC51092

[77] Golks A Tran TT Goetschy JF Guerini D. Requirement for O-linked N-acetylglucosaminyltransferase in lymphocytes activation. EMBO J. (2007) 26:4368–79. 10.1038/sj.emboj.7601845 17882263 PMC2034663

[78] Woo CM Lund PJ Huang AC Davis MM Bertozzi CR Pitteri SJ. Mapping and quantification of over 2000 O-linked glycopeptides in activated human T cells with isotope-targeted glycoproteomics (Isotag). Mol Cell Proteomics (2018) 17:764–75. 10.1074/mcp.RA117.000261 29351928 PMC5880114

[79] Li B Kohler JJ. Glycosylation of the nuclear pore. Traffic (2014) 15:347–61. 10.1111/tra.12150 24423194 PMC4001855

[80] Wysocka J Myers MP Laherty CD Eisenman RN Herr W. Human Sin3 deacetylase and trithorax-related Set1/Ash2 histone H3-K4 methyltransferase are tethered together selectively by the cell-proliferation factor HCF-1. Genes Dev. (2003) 17:896–911. 10.1101/gad.252103 12670868 PMC196026

[81] Wang Z Pandey A Hart GW. Dynamic interplay between O-linked N-acetylglucosaminylation and glycogen synthase kinase-3-dependent phosphorylation. Mol Cell Proteomics (2007) 6:1365–79. 10.1074/mcp.M600453-MCP200 17507370

[82] Myers SA Daou S Affar el B Burlingame A. Electron transfer dissociation (ETD): the mass spectrometric breakthrough essential for O-GlcNAc protein site assignments-a study of the O-GlcNAcylated protein host cell factor C1. Proteomics (2013) 13:982–91. 10.1002/pmic.201200332 23335398 PMC3988289

[83] Fujiwara T Oda K Yokota S Takatsuki A Ikehara Y. Brefeldin A causes disassembly of the Golgi complex and accumulation of secretory proteins in the endoplasmic reticulum. J Biol Chem. (1988) 263:18545–52. 3192548

[84] Lippincott-Schwartz J Yuan LC Bonifacino JS Klausner RD. Rapid redistribution of Golgi proteins into the ER in cells treated with brefeldin A: evidence for membrane cycling from Golgi to ER. Cell (1989) 56:801–13. 10.1016/0092-8674(89)90685-5 2647301 PMC7173269

[85] Peyroche A Antonny B Robineau S Acker J Cherfils J Jackson CL. Brefeldin A Acts to stabilize an abortive ARF–GDP–Sec7 domain protein complex involvement of specific residues of the sec7 domain. Mol Cell (1999) 3:275–85. 10.1016/S1097-2765(00)80455-4 10198630

[86] Staub O Dho S Henry P Correa J Ishikawa T McGlade J. WW domains of Nedd4 bind to the proline-rich PY motifs in the epithelial Na+ channel deleted in Liddle's syndrome. EMBO J. (1996) 15:2371–80. 10.1002/j.1460-2075.1996.tb00593.x 8665844 PMC450167

[87] Staub O Gautschi I Ishikawa T Breitschopf K Ciechanover A Schild L. Regulation of stability and function of the epithelial Na+ channel (ENaC) by ubiquitination. EMBO J. (1997) 16:6325–36. 10.1093/emboj/16.21.6325 9351815 PMC1170239

[88] Watson PJ Frigerio G Collins BM Duden R Owen DJ. Gamma-COP appendage domain - structure and function. Traffic (2004) 5:79–88. 10.1111/j.1600-0854.2004.00158.x 14690497

[89] Frigerio G Grimsey N Dale M Majoul I Duden R. Two human ARFGAPs associated with COP-I-coated vesicles. Traffic (2007) 8:1644–55. 10.1111/j.1600-0854.2007.00631.x 17760859 PMC2171037

[90] Bonifacino JS Hurley JH. Retromer. Curr Opin Cell Biol. (2008) 20:427–36. 10.1016/j.ceb.2008.03.009 18472259 PMC2833274

[91] Granata A Schiavo G Warner TT. TorsinA and dystonia: from nuclear envelope to synapse. J Neurochem. (2009) 109:1596–609. 10.1111/j.1471-4159.2009.06095.x 19457118

[92] Kliouchnikov L Bigay J Mesmin B Parnis A Rawet M Goldfeder N. Discrete determinants in ArfGAP2/3 conferring Golgi localization and regulation by the COPI coat. Mol Biol Cell (2009) 20:859–69. 10.1091/mbc.e08-10-1010 19109418 PMC2633400

[93] Granata A Warner TT. The role of torsinA in dystonia. Eur J Neurol. (2010) 17(Suppl. 1):81–7. 10.1111/j.1468-1331.2010.03057.x 20590813

[94] Laney JD Hochstrasser M Analysis of protein ubiquitination. Curr Protoc Protein Sci Chapter (2011) 14:15 10.1002/0471140864.ps1405s66 22045559

[95] Zaro BW Yang YY Hang HC Pratt MR. Chemical reporters for fluorescent detection and identification of O-GlcNAc-modified proteins reveal glycosylation of the ubiquitin ligase NEDD4-1. Proc Natl Acad Sci USA. (2011) 108:8146–51. 10.1073/pnas.1102458108 21540332 PMC3100932

[96] Boase NA Kumar S. NEDD4: The founding member of a family of ubiquitin-protein ligases. Gene (2015) 557:113–22. 10.1016/j.gene.2014.12.020 25527121 PMC6639052

[97] Goel P Manning JA Kumar S. NEDD4-2 (NEDD4L): The ubiquitin ligase for multiple membrane proteins. Gene (2015) 557:1–10. 10.1016/j.gene.2014.11.051 25433090 PMC6636357

[98] Wang S Bellen HJ. The retromer complex in development and disease. Development (2015) 142:2392–6. 10.1242/dev.123737 26199408 PMC4510866

[99] Laudermilch E Schlieker C. Torsin ATPases: structural insights and functional perspectives. Curr Opin Cell Biol. (2016) 40:1–7. 10.1016/j.ceb.2016.01.001 26803745 PMC4887320

[100] Verges M. Retromer in polarized protein transport. Int Rev Cell Mol Biol. (2016) 323:129–79. 10.1016/bs.ircmb.2015.12.005 26944621

[101] Williams ET Chen X Moore DJ. VPS35, the retromer complex and Parkinson's Disease. J Parkinsons Dis. (2017) 7:219–33. 10.3233/JPD-161020 28222538 PMC5438477

[102] Waters MG Serafini T Rothman JE. 'Coatomer': a cytosolic protein complex containing subunits of non-clathrin-coated Golgi transport vesicles. Nature (1991) 349:248–51. 10.1038/349248a0 1898986

[103] Hara-Kuge S Kuge O Orci L Amherdt M Ravazzola M Wieland FT. En bloc incorporation of coatomer subunits during the assembly of COP-coated vesicles. J Cell Biol. (1994) 124:883–92. 10.1083/jcb.124.6.883 8132710 PMC2119964

[104] Gaynor EC Graham TR Emr SD. COPI in ER/Golgi and intra-Golgi transport: do yeast COPI mutants point the way? Biochim Biophys Acta (1998) 1404:33–51. 10.1016/S0167-4889(98)00045-7 9714721

[105] Strating JR Martens GJ. The p24 family and selective transport processes at the ER-Golgi interface. Biol Cell (2009) 101:495–509. 10.1042/BC20080233 19566487

[106] Sheff D Lowe M Kreis TE Mellman I. Biochemical heterogeneity and phosphorylation of coatomer subunits. J Biol Chem. (1996) 271:7230–6. 10.1074/jbc.271.12.7230 8636162

[107] Christensen GL Kelstrup CD Lyngso C Sarwar U Bogebo R Sheikh SP. Quantitative phosphoproteomics dissection of seven-transmembrane receptor signaling using full and biased agonists. Mol Cell Proteomics (2010) 9:1540–53. 10.1074/mcp.M900550-MCP200 20363803 PMC2938087

[108] Kim W Bennett EJ Huttlin EL Guo A Li J Possemato A. Systematic and quantitative assessment of the ubiquitin-modified proteome. Mol Cell (2011) 44:325–40. 10.1016/j.molcel.2011.08.025 21906983 PMC3200427

[109] Imami K Sugiyama N Imamura H Wakabayashi M Tomita M Taniguchi M. Temporal profiling of lapatinib-suppressed phosphorylation signals in EGFR/HER2 pathways. Mol Cell Proteomics (2012) 11:1741–57. 10.1074/mcp.M112.019919 22964224 PMC3518135

[110] Mertins P Qiao JW Patel J Udeshi ND Clauser KR Mani DR. Integrated proteomic analysis of post-translational modifications by serial enrichment. Nat Methods (2013) 10:634–7. 10.1038/nmeth.2518 23749302 PMC3943163

[111] Schweppe DK Rigas JR Gerber SA. Quantitative phosphoproteomic profiling of human non-small cell lung cancer tumors. J Proteomics (2013) 91:286–96. 10.1016/j.jprot.2013.07.023 23911959 PMC3825743

[112] Mertins P Yang F Liu T Mani DR Petyuk VA Gillette MA Ischemia in tumors induces early and sustained phosphorylation changes in stress kinase pathways but does not affect global protein levels. Mol Cell Proteomics (2014) 13:1690–704. 10.1074/mcp.M113.036392 24719451 PMC4083109

[113] Sharma K D'Souza RC Tyanova S Schaab C Wisniewski JR Cox J. Ultradeep human phosphoproteome reveals a distinct regulatory nature of Tyr and Ser/Thr-based signaling. Cell Rep. (2014) 8:1583–94. 10.1016/j.celrep.2014.07.036 25159151

[114] Stuart SA Houel S Lee T Wang N Old WM Ahn NG. A phosphoproteomic comparison of B-RAFV600E and MKK1/2 inhibitors in melanoma cells. Mol Cell Proteomics (2015) 14:1599–615. 10.1074/mcp.M114.047233 25850435 PMC4458723

[115] Larsen SC Sylvestersen KB Mund A Lyon D Mullari M Madsen MV. Proteome-wide analysis of arginine monomethylation reveals widespread occurrence in human cells. Sci Signal. (2016) 9:rs9. 10.1126/scisignal.aaf7329 27577262

[116] Mertins P Mani DR Ruggles KV Gillette MA Clauser KR Wang P. Proteogenomics connects somatic mutations to signalling in breast cancer. Nature (2016) 534:55–62. 10.1038/nature18003 27251275 PMC5102256

[117] Dodonova SO Diestelkoetter-Bachert P von Appen A Hagen WJ Beck R Beck M. VESICULAR TRANSPORT. A structure of the COPI coat and the role of coat proteins in membrane vesicle assembly. Science (2015) 349:195–8. 10.1126/science.aab1121 26160949

[118] Ramakrishnan B Qasba PK. Structure-based design of beta 1,4-galactosyltransferase I (beta 4Gal-T1) with equally efficient N-acetylgalactosaminyltransferase activity: point mutation broadens beta 4Gal-T1 donor specificity. J Biol Chem. (2002) 277:20833–9. 10.1074/jbc.M111183200 11916963

[119] Wells L Vosseller K Cole RN Cronshaw JM Matunis MJ Hart GW. Mapping sites of O-GlcNAc modification using affinity tags for serine and threonine post-translational modifications. Mol Cell Proteomics (2002) 1:791–804. 10.1074/mcp.M200048-MCP200 12438562

[120] Khidekel N Arndt S Lamarre-Vincent N Lippert A Poulin-Kerstien KG Ramakrishnan B. A chemoenzymatic approach toward the rapid and sensitive detection of O-GlcNAc posttranslational modifications. J Am Chem Soc. (2003) 125:16162–3. 10.1021/ja038545r 14692737

[121] Tai HC Khidekel N Ficarro SB Peters EC Hsieh-Wilson LC. Parallel identification of O-GlcNAc-modified proteins from cell lysates. J Am Chem Soc. (2004) 126:10500–1. 10.1021/ja047872b 15327282

[122] Vosseller K Hansen KC Chalkley RJ Trinidad JC Wells L Hart GW Quantitative analysis of both protein expression and serine / threonine post-translational modifications through stable isotope labeling with dithiothreitol. Proteomics (2005) 5:388–98. 10.1002/pmic.200401066 15648052

[123] Khidekel N Ficarro SB Clark PM Bryan MC Swaney DL Rexach JE. Probing the dynamics of O-GlcNAc glycosylation in the brain using quantitative proteomics. Nat Chem Biol. (2007) 3:339–48. 10.1038/nchembio881 17496889

[124] Clark PM Dweck JF Mason DE Hart CR Buck SB Peters EC. Direct in-gel fluorescence detection and cellular imaging of O-GlcNAc-modified proteins. J Am Chem Soc. (2008) 130:11576–7. 10.1021/ja8030467 18683930 PMC2649877

[125] Chalkley RJ Thalhammer A Schoepfer R Burlingame AL. Identification of protein O-GlcNAcylation sites using electron transfer dissociation mass spectrometry on native peptides. Proc Natl Acad Sci USA. (2009) 106:8894–9. 10.1073/pnas.0900288106 19458039 PMC2690010

[126] Rexach JE Rogers CJ Yu SH Tao J Sun YE Hsieh-Wilson LC. Quantification of O-glycosylation stoichiometry and dynamics using resolvable mass tags. Nat Chem Biol. (2010) 6:645–51. 10.1038/nchembio.412 20657584 PMC2924450

[127] Sakabe K Wang Z Hart GW. Beta-N-acetylglucosamine (O-GlcNAc) is part of the histone code. Proc Natl Acad Sci USA. (2010) 107:19915–20. 10.1073/pnas.1009023107 21045127 PMC2993388

[128] Wang Z Udeshi ND O'Malley M Shabanowitz J Hunt DF Hart GW. Enrichment and site mapping of O-linked N-acetylglucosamine by a combination of chemical/enzymatic tagging, photochemical cleavage, and electron transfer dissociation mass spectrometry. Mol Cell Proteomics (2010) 9:153–60. 10.1074/mcp.M900268-MCP200 19692427 PMC2808261

[129] Myers SA Panning B Burlingame AL. Polycomb repressive complex 2 is necessary for the normal site-specific O-GlcNAc distribution in mouse embryonic stem cells. Proc Natl Acad Sci USA. (2011) 108:9490–5. 10.1073/pnas.1019289108 21606357 PMC3111310

[130] Alfaro JF Gong CX Monroe ME Aldrich JT Clauss TR Purvine SO. Tandem mass spectrometry identifies many mouse brain O-GlcNAcylated proteins including EGF domain-specific O-GlcNAc transferase targets. Proc Natl Acad Sci USA. (2012) 109:7280–5. 10.1073/pnas.1200425109 22517741 PMC3358849

[131] Rexach JE Clark PM Mason DE Neve RL Peters EC Hsieh-Wilson LC. Dynamic O-GlcNAc modification regulates CREB-mediated gene expression and memory formation. Nat Chem Biol. (2012) 8:253–61. 10.1038/nchembio.770 22267118 PMC3288555

[132] Ma J Hart GW. O-GlcNAc profiling: from proteins to proteomes. Clinical Proteomics (2014) 11:1–16. 10.1186/1559-0275-11-8 24593906 PMC4015695

[133] Ma J Liu T Wei AC Banerjee P O'Rourke B Hart GW. O-GlcNAcomic profiling identifies widespread O-Linked beta-N-Acetylglucosamine modification (O-GlcNAcylation) in oxidative phosphorylation system regulating cardiac mitochondrial function. J Biol Chem. (2015) 290:29141–53. 10.1074/jbc.M115.691741 26446791 PMC4705920

[134] Cox NJ Meister TR Boyce M Chemical biology of O-GlcNAc glycosylation. In: Wang L-X Tan Z, editors. Chemical Biology of Glycoproteins. Cambridge: Royal Society of Chemistry (2017). p. 94–149.

[135] Langer JD Roth CM Béthune J Stoops EH Brügger B Herten DP. A Conformational Change in the α-subunit of Coatomer Induced by Ligand Binding to γ-COP Revealed by Single-pair FRET. Traffic (2008) 9:597–607. 10.1111/j.1600-0854.2007.00697.x 18182008

[136] Reinhard C Harter C Bremser M Brugger B Sohn K Helms JB. Receptor-induced polymerization of coatomer. Proc Natl Acad Sci USA. (1999) 96:1224–8. 10.1073/pnas.96.4.1224 9990005 PMC15444

[137] Deng RP He X Guo SJ Liu WF Tao Y Tao SC. Global identification of O-GlcNAc transferase (OGT) interactors by a human proteome microarray and the construction of an OGT interactome. Proteomics (2014) 14:1020–30. 10.1002/pmic.201300144 24536041

[138] Tarbet HJ Toleman CA Boyce M. A sweet embrace: control of protein-protein interactions by O-linked beta-N-acetylglucosamine. Biochemistry (2018) 57:13–21. 10.1021/acs.biochem.7b00871 29099585 PMC5807727

[139] Fiedler K Veit M Stamnes MA Rothman JE. Bimodal interaction of coatomer with the p24 family of putative cargo receptors. Science (1996) 273:1396–9. 10.1126/science.273.5280.1396 8703076

[140] Sohn K Orci L Ravazzola M Amherdt M Bremser M Lottspeich F. A major transmembrane protein of Golgi-derived COPI-coated vesicles involved in coatomer binding. J Cell Biol. (1996) 135:1239–48. 10.1083/jcb.135.5.1239 8947548 PMC2121093

[141] Bethune J Kol M Hoffmann J Reckmann I Brugger B Wieland F. Coatomer, the coat protein of COPI transport vesicles, discriminates endoplasmic reticulum residents from p24 proteins. Mol Cell Biol. (2006) 26:8011–21. 10.1128/MCB.01055-06 16940185 PMC1636745

[142] Bykov YS Schaffer M Dodonova SO Albert S Plitzko JM Baumeister W. The structure of the COPI coat determined within the cell. Elife (2017) 6:e32493. 10.7554/eLife.32493 29148969 PMC5716667

[143] Dodonova SO Aderhold P Kopp J Ganeva I Rohling S Hagen WJH. 9A structure of the COPI coat reveals that the Arf1 GTPase occupies two contrasting molecular environments. Elife (2017) 6:e266691. 10.7554/eLife.26691 28621666 PMC5482573

[144] Hornbeck PV Zhang B Murray B Kornhauser JM Latham V Skrzypek E. PhosphoSitePlus, 2014: mutations, PTMs and recalibrations. Nucleic Acids Res. (2015) 43(Database issue):D512–20. 10.1093/nar/gku1267 25514926 PMC4383998

[145] Wang Z Udeshi ND Slawson C Compton PD Sakabe K Cheung WD. Extensive crosstalk between O-GlcNAcylation and phosphorylation regulates cytokinesis. Sci Signal. (2010) 3:ra2. 10.1126/scisignal.2000526 20068230 PMC2866299

[146] Trinidad JC Barkan DT Gulledge BF Thalhammer A Sali A Schoepfer R. Global identification and characterization of both O-GlcNAcylation and phosphorylation at the murine synapse. Mol Cell Proteomics (2012) 11:215–29. 10.1074/mcp.O112.018366 22645316 PMC3412957

[147] Wang S Huang X Sun D Xin X Pan Q Peng S. Extensive crosstalk between O-GlcNAcylation and phosphorylation regulates Akt signaling. PLoS ONE (2012) 7:e37427. 10.1371/journal.pone.0037427 22629392 PMC3358304

[148] Zhong J Martinez M Sengupta S Lee A Wu X Chaerkady R. Quantitative phosphoproteomics reveals crosstalk between phosphorylation and O-GlcNAc in the DNA damage response pathway. Proteomics (2015) 15:591–607. 10.1002/pmic.201400339 25263469 PMC4564869

[149] Leney AC El Atmioui D Wu W Ovaa H Heck AJR. Elucidating crosstalk mechanisms between phosphorylation and O-GlcNAcylation. Proc Natl Acad Sci USA. (2017) 114:201620529. 10.1073/pnas.1620529114 28808029 PMC5584407

