# Supplementary material for: A Novel Glycoproteomics Workflow Reveals Dynamic O-GlcNAcylation of COPγ1 as a Candidate Regulator of Protein Trafficking
Source: Front Endocrinol (Lausanne). 2018 Oct 15;9:606. doi: 10.3389/fendo.2018.00606 (PMC6232944; doi:10.3389/fendo.2018.00606)
Supplement: Supplementary file 1 [file Table_1.pdf]

COPγ1 O-GlcNAc MS (Table 2): Complete data set for COPγ1 O-GlcNAc site-mapping. Two biological replicates are included. All replicates include a complete peptide list and O-GlcNAc-modified peptide list.

SILAC BFA data (Table 3): Complete data set for BFA glycoproteomics workflow. Contains lists of proteins with concordant BFA-dependent changes across biological replicates that also have a fold-change magnitude greater than 2 (nuclear fractions) or 1.5 (cytoplasmic fractions).

Alkyne bead enrichment test (Table 4): Total spectral counts for each protein identified in proof of principle alkyne-bead click enrichment test. Complete data set for Figure 2.

SILAC heavy incorporation test (Table 5): Peptide data from SILAC incorporation test. Heavy arginine (Label:$^{13}C_8{}^{15}N_2$ (+10.02) and heavy lysine (Label:$^{13}C_6{}^{15}N_2$ (+8.01)) were searched as variable modifications and found in every non-contaminant protein.
